# Supplementary material for: The immune cell atlas of human neuroblastoma
Source: Cell Rep Med. 2022 Jun 9;3(6):100657. doi: 10.1016/j.xcrm.2022.100657 (PMC9245004; doi:10.1016/j.xcrm.2022.100657)
Supplement: Document S1. Figures S1–S5 [file mmc1.pdf]

Cell Reports Medicine, Volume *3*

Supplemental information

The immune cell atlas of human neuroblastoma

Bronte Manouk Verhoeven, Shenglin Mei, Thale Kristin Olsen, Karin Gustafsson, Anders Valind, Axel Lindström, David Gisselsson, Shahrzad Shirazi Fard, Catharina Hagerling, Peter V. Kharchenko, Per Kogner, John Inge Johnsen, and Ninib Baryawno

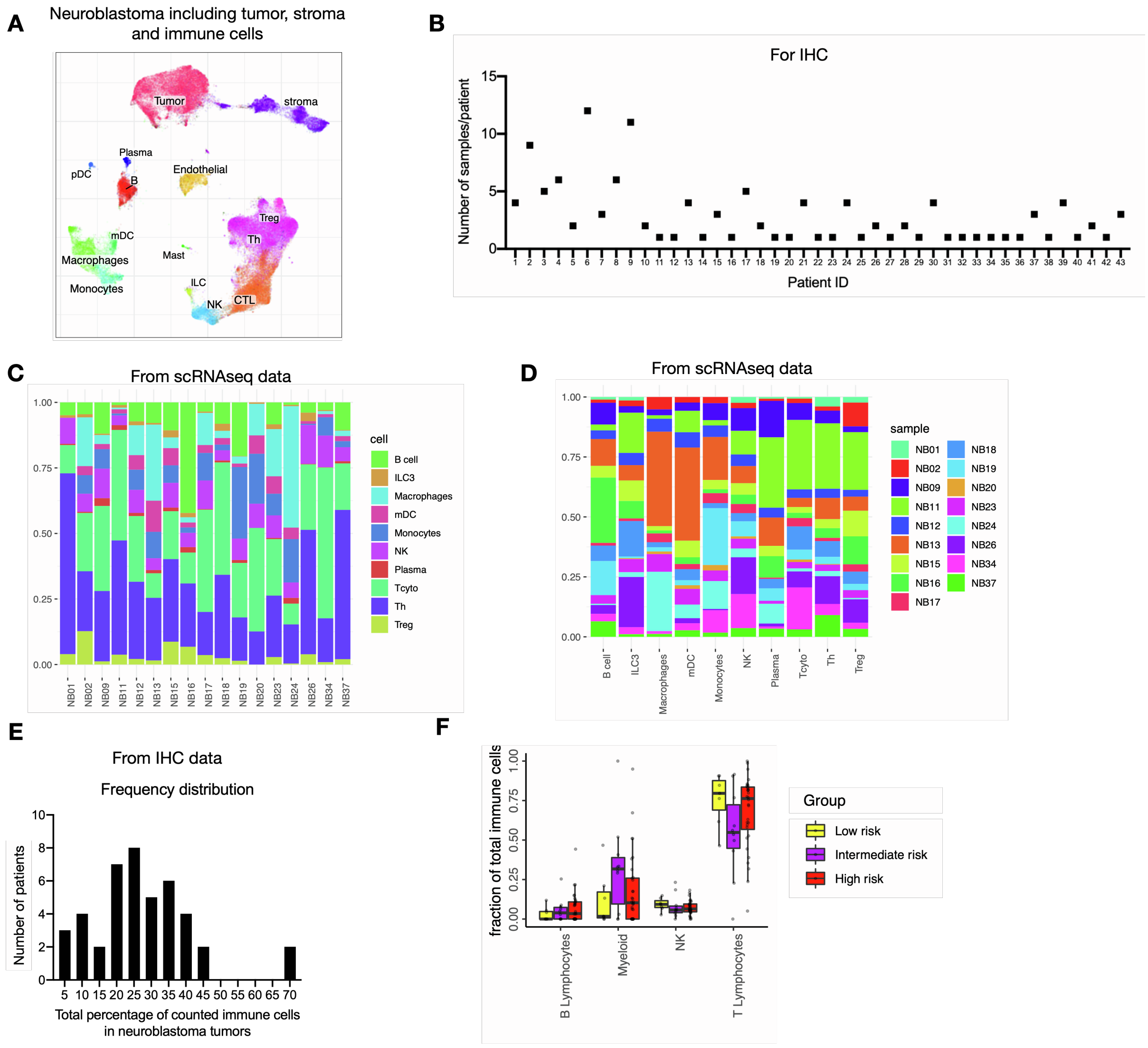

Figure S1. Global immune cell landscape of human NB. Related to Figure 1. (**A**) Global overview of neuroblastoma landscape including tumor and stromal cells from 19 patient samples. (**B**) The number of samples taken per patient used for the immunohistochemistry analysis. (**C**) The number of the different immune cell populations per patient sample (n=17) (from scRNAseq data). (**D**) Barplot showing cell composition of different immune cell types per patient sample (from scRNAseq data). (**E**) Frequency distribution of total percentage of counted immune cells in neuroblastoma tumors (from IHC data) shown as barplot (n=43). (**F**) Fraction of immune cell populations in low- (n=8), intermediate- (n=10) and high-risk (n=34) patient samples from the combined dataset including Kildisiute et al. Science Advances 2021, Dong et al. Cancer Cell 2020, Kameneva et al. Nature Genetics 2021 and the data presented here.

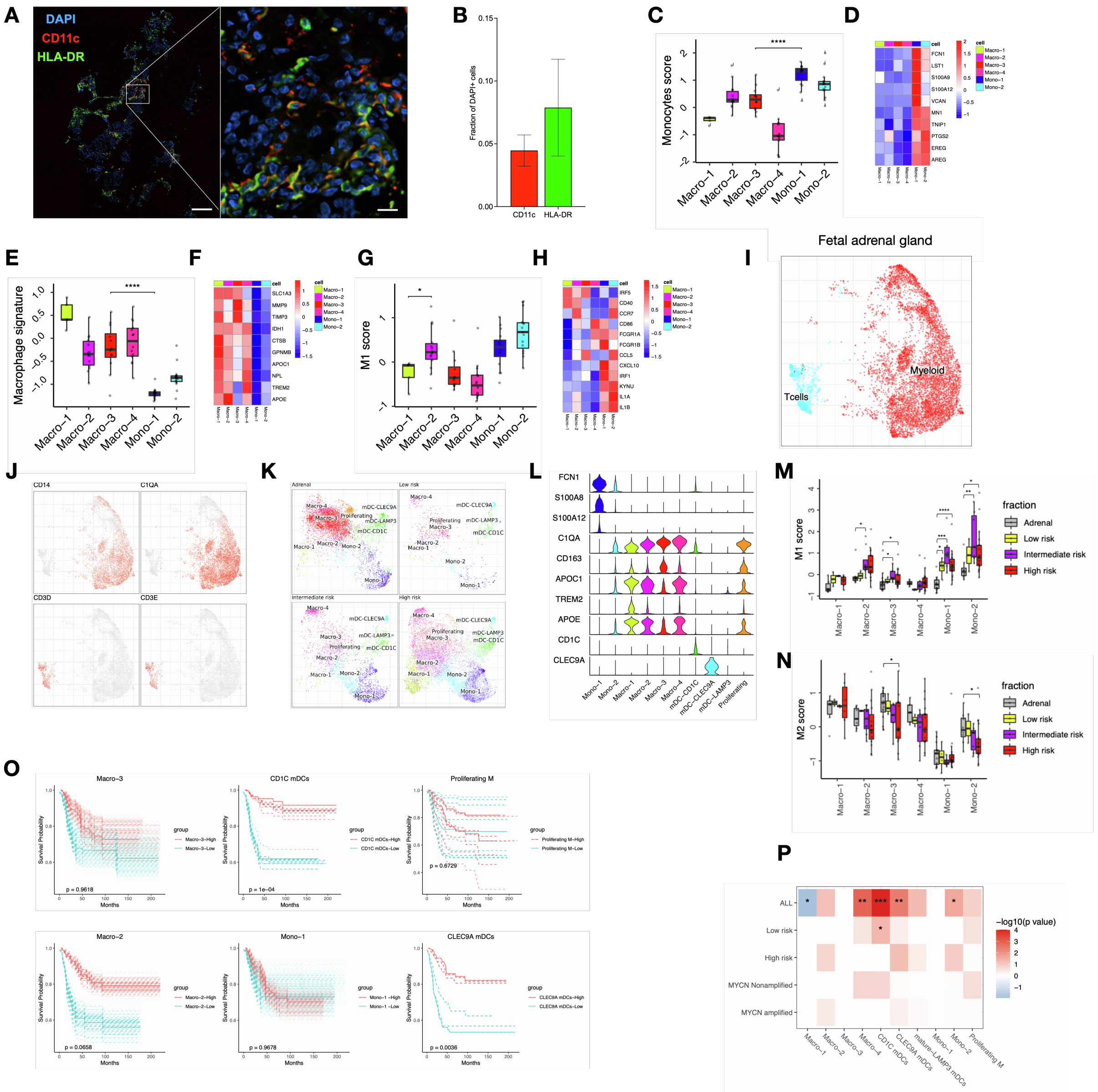

Figure S2. Myeloid cell infiltration with distinct cell-states detected in NB. Related to Figure 2. (**A**) Infiltration of CD11c+ myeloid cells and antigen presentation function (HLA-DR+) into neuroblastoma tumor using multiplex immunohistochemistry. Scale bar left = 800 μm, scale bar right = 50 μm. Picture taken from patient sample NB06. (**B**) Quantification for panel A presented as mean with standard error of the mean (n=3). (**C-D**) Average expression of monocyte score signature and accompanying heat map (n=16) (top color bar, colors matching panel C). Wilcoxon rank sum test was used for statistical analysis, *p<0.05, **p<0.01, ***p<0.001, ****p<0.0001. (**E-F**) Average expression of macrophage signature genes in different myeloid subpopulations and accompanying heat map (n=16) (top color bar, colors matching panel E). Wilcoxon rank sum test was used for statistical analysis, *p<0.05, **p<0.01, ***p<0.001, ****p<0.0001. (**G-H**) Average expression of M1 signature genes in different myeloid subpopulations and accompanying heat map (n=16) (top color bar, colors matching panel G). Wilcoxon rank sum test was used for statistical analysis, *p<0.05. (**I**) UMAP visualization of fetal adrenal immune cell data. (**J**) Accompanying key marker genes for I. (**K**) Myeloid cell specific embedding shows different numbers of different myeloid cell subpopulations comparing low- (n=5), intermediate- (n=8) and high-risk (n=21) neuroblastoma. (**L**) Violin plot displaying specific gene expression per population. Average expression of M1 (**M**) and M2 (**N**) signature genes in the combined dataset (see K for biological replicate numbers). Wilcoxon rank sum test was used for statistical analysis, *p<0.05, **p<0.01, ***p<0.001, ****p<0.0001. (**O**) Similar to Figure 1H, survival curves for Macro-2, Macro-3, Mono-1, CD1C mDCs, mature-LAMP3 mDCs, CLEC9A mDCs and Proliferating myeloid cells. (**P**) Summary of survival correlation split into risk group and MYCN status for the myeloid subtypes. Log-rank test was used for statistical analysis, *p<0.05, **p<0.01, ***p<0.001, ****p<0.0001.

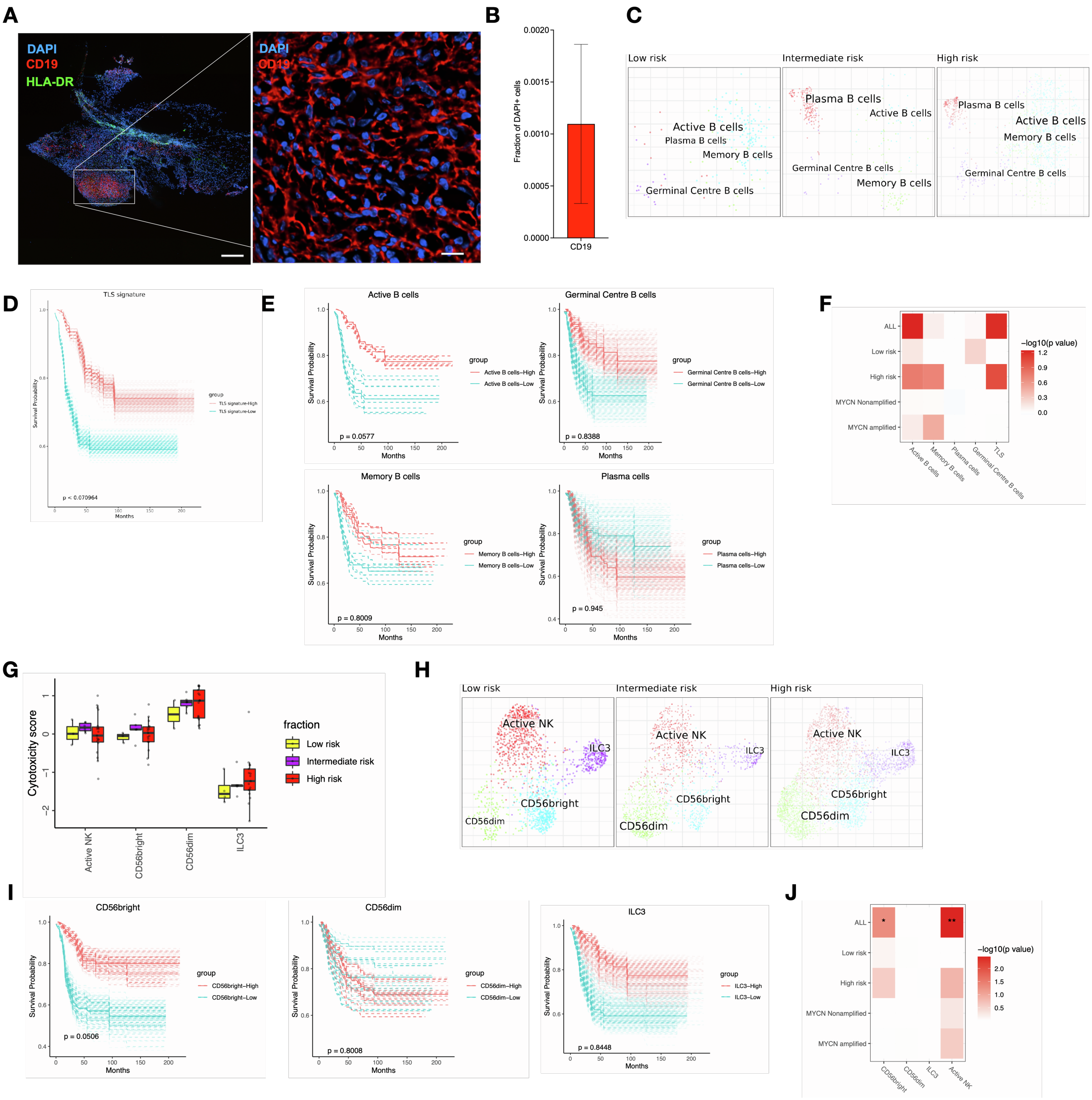

Figure S3. B and NK cell heterogeneity and infiltration in NB tumors. Related to Figure 3. (**A**) B cell (CD19+) and antigen presentation (HLA-DR+) staining using multiplex immunohistochemistry. Scale bar left = 1600 µm, scale bar right = 50 µm. Picture taken from patient sample NB11. (**B**) Quantification for panel A presented as mean with standard error of the mean (n=4). (**C**) B cell specific embedding shows different numbers of different B cell subpopulations comparing low- (n=3), intermediate- (n=6) and high-risk (n=18) neuroblastoma. (**D**) Similar to Figure 1H, survival curve for TLS signature. (**E**) Survival curves for the different B cell subpopulations. (**F**) Summary of survival correlation split into risk group and MYCN status for the B cell subtypes. Log-rank test was used for statistical analysis, $*p<0.05$, $**p<0.01$, $***p<0.001$, $****p<0.0001$.(**G**) Cytotoxicity signature score for the different NK cell populations comparing low- (n=4), intermediate- (n=6) and high-risk (n=21) disease. (**H**) The fraction of NK cells in low- (n=4), intermediate- (n=6) and high-risk (n=21) neuroblastoma on an NK cell specific embedding. (**I**) Survival curves for CD56bright, CD56dim and ILC3 cells. (**J**) Summary of survival correlation split into risk group and MYCN status for the NK cell subtypes. Log-rank test was used for statistical analysis, $*p<0.05$, $**p<0.01$, $***p<0.001$, $****p<0.0001$.

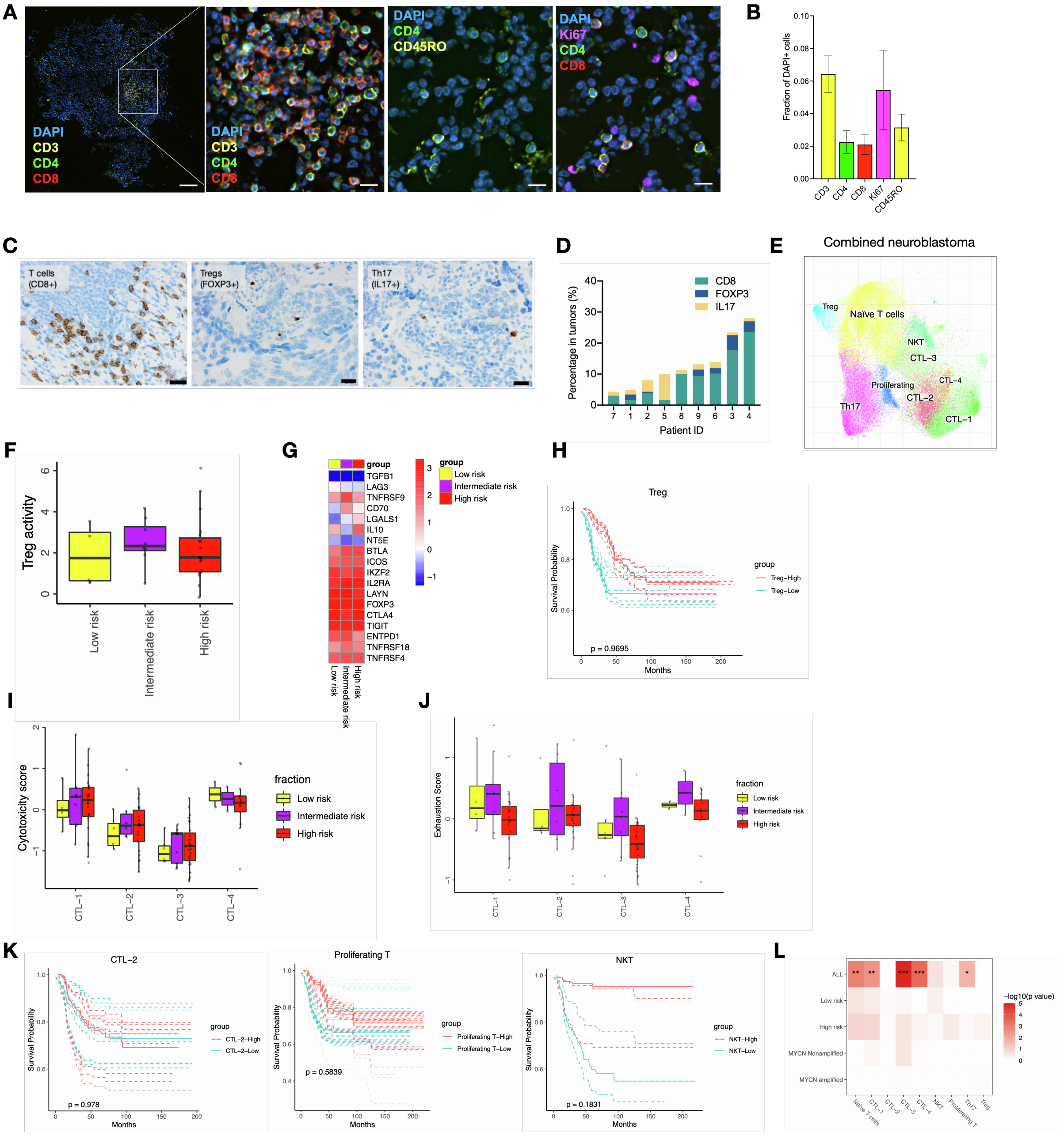

Figure S4. Distinct subtypes of T cell infiltrates correlate with improved NB survival. Related to Figure 4. (**A**) Multiplex IHC validation of different T cell populations in human neuroblastoma. Scale bar left = 800 µm, further right scale bars all are 50 µm. Staining included CD3+, CD4+, CD8+, memory cells (CD45RO+) and proliferating cells (Ki67+), and (**B**) quantification is presented as mean with standard error of the mean (n=3 to n=5). Pictures taken from patient sample NB01. (**C-D**) Additional single staining for CD8, FOXP3 and IL-17, and quantifications (n=9). (**E**) T cell specific embedding for the T cells present in the combined dataset (as used for the myeloid cell analysis). (**F**) Treg activity signature score comparing low- (n=4), intermediate- (n=9) and high-risk (n=23) disease with accompanying heat map (**G**). (**H**) Survival curve for Tregs. (**I-J**) Cytotoxicity and exhaustion signature score comparing low- (n=4), intermediate- (n=9) and high-risk (n=23) neuroblastoma for CTL-1 to -4. (**K**) Survival curves for CTL-2, proliferating T cells and NKT cells. (**L**) Summary of survival correlation split into risk group and MYCN status for the T cell subtypes. Log-rank test was used for statistical analysis, *p<0.05, **p<0.01, ***p<0.001, ****p<0.0001.

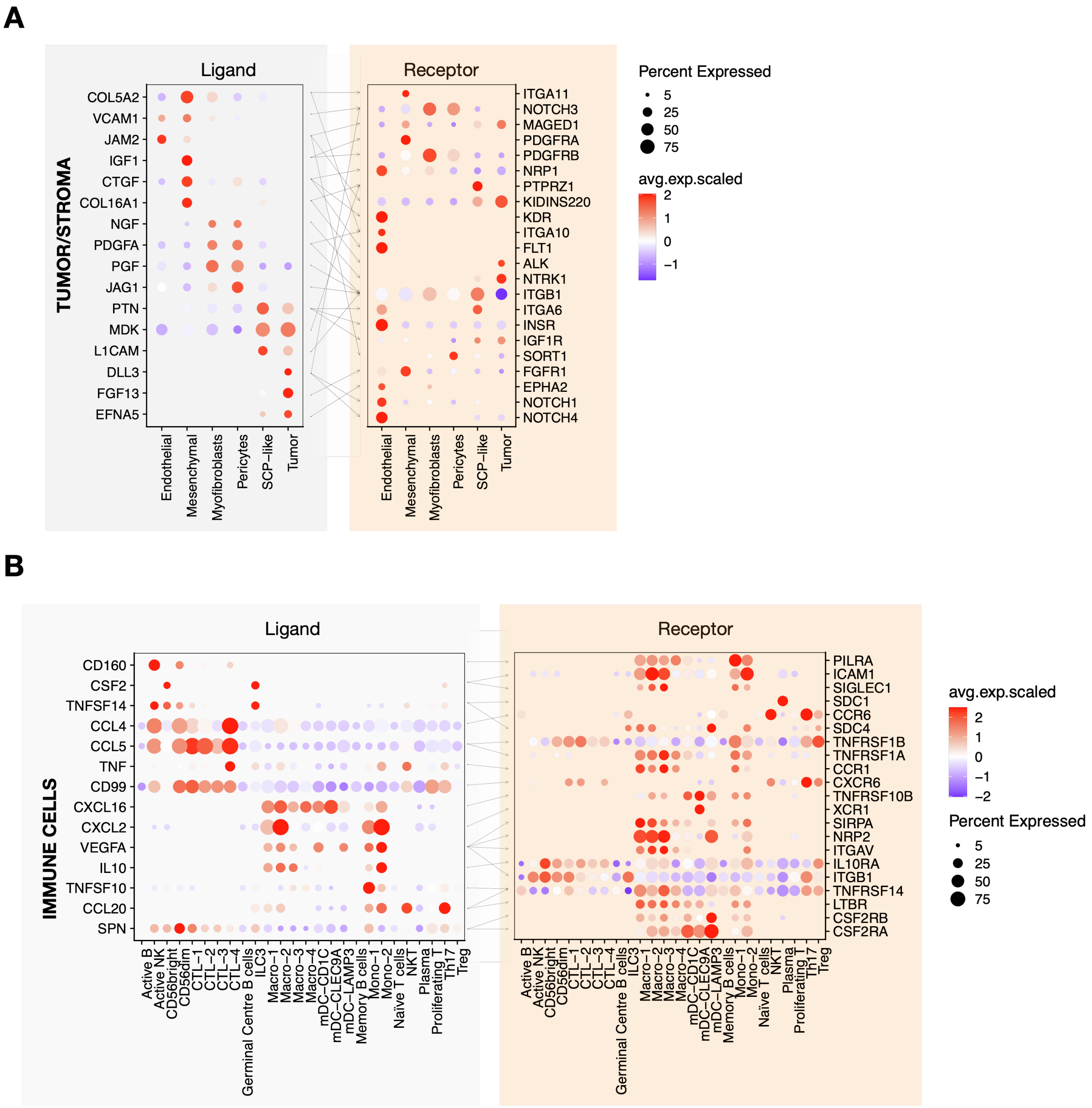

Figure S5. Tumor-immune cell ligand-receptor interaction analysis reveals several interactions for future studies. Related to Figure 5. (**A**) Similar to Figure 5C, showing expression of Ligand (tumor/stroma) - Receptor (tumor/stroma) interactions (n=19). (**B**) Ligand (immune) - Receptor (immune) interactions between immune cell subsets. The color represents scaled average expression of marker genes in each cell type, and the size indicates the proportion of cells expressing marker genes. Significance of ligand receptor pair is determined by permutation test, correlation to survival and specific cellular expression (see method).
