# Supplementary material for: The immune cell atlas of human neuroblastoma
Source: Cell Rep Med. 2022 Jun 9;3(6):100657. doi: 10.1016/j.xcrm.2022.100657 (PMC9245004; doi:10.1016/j.xcrm.2022.100657)
Supplement: Document S2. Article plus supplemental information [file mmc7.pdf]

Cell Reports Medicine

Article

The immune cell atlas of human neuroblastoma

Graphical abstract

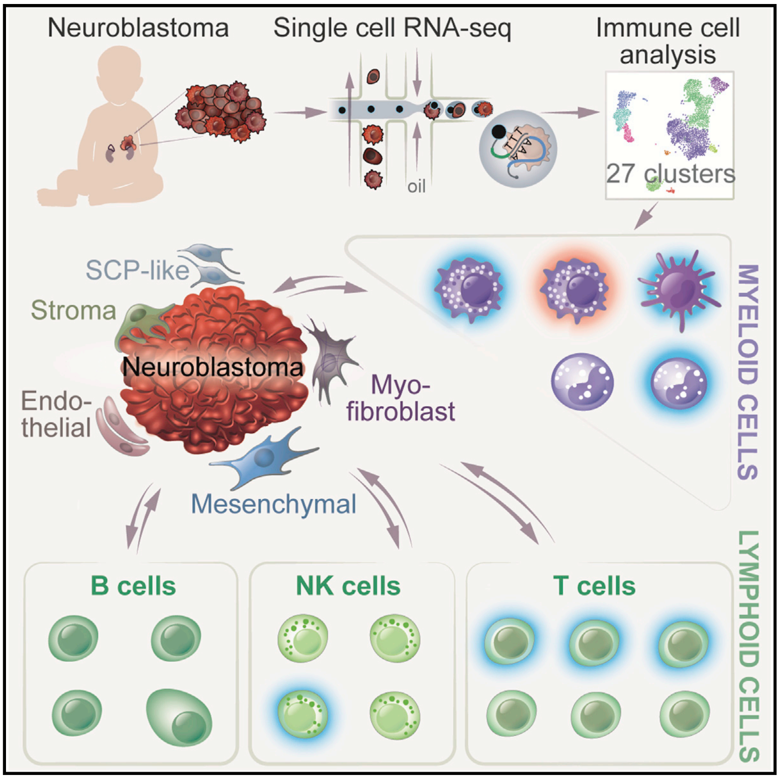

Authors

Bronte Manouk Verhoeven, Shenglin Mei, Thale Kristin Olsen, ..., Per Kogner, John Inge Johnsen, Ninib Baryawno

Correspondence

n.baryawno@ki.se

In brief

Verhoeven et al. provide a detailed description of 27 different immune cell subtypes in human neuroblastoma. Several subtypes show functional properties and correlate with improved survival. Together with tumor/stromal cells, the immune cells form a highly complex and interactive neuroblastoma tumor microenvironment where several interactions are proposed for future therapies.

Highlights

- 27 different immune subtypes form a complex neuroblastoma microenvironment
- Inflammatory monocytes correlate with neuroblastoma disease progression
- Active NK cells correlate with improved survival in human neuroblastoma
- T cell exhaustion and Treg activity demonstrate no difference between risk groups

Verhoeven et al., 2022, Cell Reports Medicine *3*, 100657
June 21, 2022 © 2022 The Author(s).
https://doi.org/10.1016/j.xcrm.2022.100657

CellPress

Cell Reports Medicine

CellPress
OPEN ACCESS

Article

The immune cell atlas of human neuroblastoma

Bronte Manouk Verhoeven,[1,6] Shenglin Mei,[2,6] Thale Kristin Olsen,[1] Karin Gustafsson,[3] Anders Valind,[4,5] Axel Lindström,[4] David Gisselsson,[4] Shahrzad Shirazi Fard,[1] Catharina Hagerling,[4,7] Peter V. Kharchenko,[2,7] Per Kogner,[1,7] John Inge Johnsen,[1,7] and Ninib Baryawno[1,7,8,*]

[1]Childhood Cancer Research Unit, Department of Women's and Children's Health, Karolinska Institutet, 171 77 Stockholm, Sweden
[2]Department of Biomedical Informatics, Harvard Medical School, Boston, MA 02115, USA
[3]Center for Regenerative Medicine, Massachusetts General Hospital, Boston, MA, USA
[4]Department of Laboratory Medicine, Division of Clinical Genetics, Lund University, 221 85 Lund, Sweden
[5]Department of Pediatrics, Skåne University Hospital, Lund, Sweden
[6]These authors contributed equally
[7]Senior author
[8]Lead contact
*Correspondence: n.baryawno@ki.se
https://doi.org/10.1016/j.xcrm.2022.100657

SUMMARY

Understanding the complete immune cell composition of human neuroblastoma (NB) is crucial for the development of immunotherapeutics. Here, we perform single-cell RNA sequencing (scRNA-seq) on 19 human NB samples coupled with multiplex immunohistochemistry, survival analysis, and comparison with normal fetal adrenal gland data. We provide a comprehensive immune cell landscape and characterize cell-state changes from normal tissue to NB. Our analysis reveals 27 immune cell subtypes, including distinct subpopulations of myeloid, NK, B, and T cells. Several different cell types demonstrate a survival benefit. In contrast to adult cancers and previous NB studies, we show an increase in inflammatory monocyte cell state when contrasting normal and tumor tissue, while no differences in cytotoxicity and exhaustion score for T cells, nor in Treg activity, are observed. Our receptor-ligand interaction analysis reveals a highly complex interactive network of the NB microenvironment from which we highlight several interactions that we suggest for future therapeutic studies.

INTRODUCTION

Neuroblastoma (NB) is a pediatric cancer deriving from the neural crest during embryonic development and arises primarily in the sympathetic nervous system.[1] It has a wide range of clinical presentations and outcomes, from spontaneously regressing tumors to highly aggressive and metastatic cases. This heterogeneity is reflected by large differences in 5-year survival rates ranging from 90% to 95% for low- and intermediate-risk disease, to 40%–50% for high-risk disease.[2]

The role of the immune system is widely recognized as critical in cancer development, progression, and therapy resistance in adults. Various immune cell subsets can either directly or indirectly support or suppress tumor growth in adult cancer.[3,4] Multiple studies in NB have pointed to a critical role for the immune system in both prognosis and response to treatment. For instance, the infiltration of T cells in therapy-resistant NB is known to improve clinical outcome,[5] while the prognostic value of the specific T cell subtypes remains unclear.[6] *Ex-vivo*-expanded tumor-infiltrating lymphocytes from patients showed phenotypic heterogeneity; however, they were non-reactive toward autologous tumor cells.[7] In addition, T cell receptor sequencing on NB-derived T cells revealed clonal expansion in only a small number of untreated patients,[8–10] indicating that a tumor antigen response may occur only in a limited number of patients. Furthermore, T cell memory formation, and a suggested active tumor microenvironment, have also been described in NB,[11] implying an active inflammatory response in these tumors. However, the recently described tissue-resident T cells, which highly resemble T memory cells, demonstrated that a memory T phenotype does not have to indicate tumor antigen recognition and memory formation.[12] Other lymphocytes, such as invariant natural killer (NK) T cells have been implied to play an important role in the spontaneous regression of NB due to their prominent presence in low-risk tumors.[13] Also, NK cell marker gene and protein expression are correlated with improved prognosis,[14] and infiltration of NK cells into NB tumors has been detected by bulk RNA sequencing.[15] Furthermore, different myeloid cell subtypes play an important role in disease progression and metastasis in NB,[16,17] where they are described as highly heterogeneous.[18,19] An increase in the number of macrophages was detected in metastatic NB patient samples compared with local primary tumors,[17] and macrophages are associated with poor prognosis[20]; whereas dendritic cell gene and protein programs in NB tumors in turn have been correlated with improved prognosis.[14] In addition, the presence of myeloid-derived suppressor cells has been observed in both human and mouse NB tumors and has been associated with poor prognosis.[21,22] These insights into the function of immunity in NB have led to the development of anti-GD2 immunotherapy that is given in conjunction with conventional treatment in high-risk NBs.[23,24]

This is an open access article under the CC BY license (http://creativecommons.org/licenses/by/4.0/).

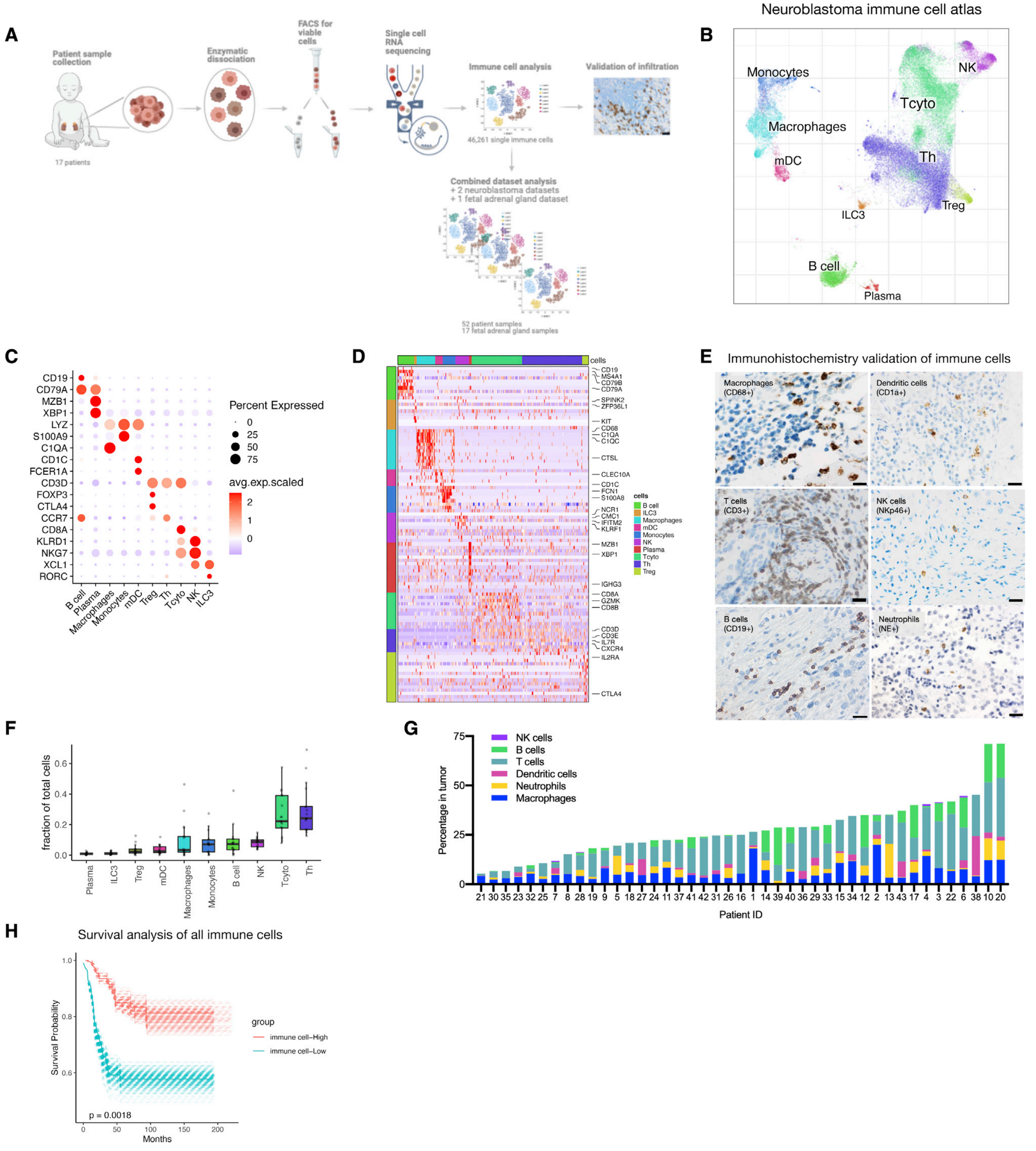

Figure 1. Global immune cell landscape of human NB

(A) Experimental design: human NB tumor tissue was mechanically and enzymatically dissociated. Immune cells were studied *in silico*, infiltration validated using immunohistochemistry, and additional data added for combinational analysis.

(B) Global overview of NB immune cell atlas containing 46,261 cells, color coded by annotated cell type (n = 17).

(C and D) (C) Subset marker gene expression and (D) heatmap of marker genes associated with major immune cell types.

(E) Images for macrophages (CD68+), dendritic cells (CD1a+), neutrophils (NE+), NK cells (NKp46+), B cells (CD19+), and T cells (CD3+) in NB tumors. Scale bar, 100 μm. n = 43.

(F) Fraction of cells from all immune cells shown for the different subtypes (n = 17).

(legend continued on next page)

CellPress
OPEN ACCESS

Although immune cell populations have been studied, many of the studies have either conveyed conflicting results, focused on a single immune cell type, or used a limited number of patient samples.[6] Previous single-cell RNA sequencing (scRNA-seq) studies in NB have mainly focused on the tumor cell compartments[25–31] and the cells in the tumor microenvironment form a complex interactive network of cells and molecules, where the immune cell function is dependent on the different cells working together in the tissue. Therefore, providing a systems biology characterization of the immune cells, their prognostic impact, and their interactions with tumor cells, and each other, at the single-cell level, will provide an improved understanding of the NB microenvironment that can be exploited for future therapies. In this study, we performed scRNA-seq on human NBs, we contrasted our data with a single-cell dataset from normal fetal adrenal tissue and additional NB datasets to provide an in-depth analysis and characterization of the different immune cells, their molecular state shifts, and cellular networks.

RESULTS

Global immune cell landscape of human NB

We performed single-cell transcriptomics (10X Chromium Single Cell Solution, see STAR Methods) on tumor tissue from 17 NB patients (19 samples). Tumor specimens were dissociated using a protocol that enriches for immune cells (Figure 1A).[32] The samples were derived from all clinical risk groups and included patients with both primary and recurrent disease (Table S1). A preprint article by our lab focused on the tumor and stroma compartment,[28] whereas in this study we focused on the immune cells of NB. We used the algorithm CONOS[33] for joint analysis of all NB samples and created an immune cell landscape (Figure 1B; for cellular map including tumor and stroma cells, see Figure S1A). Our initial analysis of the immune cells including 46,261 cells revealed a rich repertoire of the main immune subtypes, including myeloid, B, T, and NK cell lineages, and consisted of 10 clusters in total (Figures 1B–1D). The global immune cell clusters were identified using key marker genes: myeloid cells (*LYZ*, *C1QA*, *S100A9*, and *CD1C*),[34,35] B cells (*MS4A1*, *BANK1*, and *CD79A*),[36,37] T cells (*CD3D*),[38] and NK cells (*CMC1* and *GNLY*)[39] (Figures 1C and 1D). We validated immune cell infiltration of the different immune cell types detected in our scRNA-seq data by performing immunohistochemistry on a separate cohort of 43 patients (Table S2) taking one or multiple samples from the same patient (Figure S1B). We stained for key markers detecting T cells (CD3+), B cells (CD19+), NK cells (NKp46+), macrophages (CD68$^+$), dendritic cells (CD1a+), and neutrophils (NE+) (Figure 1E). Neutrophils were absent in the scRNA-seq data, reflecting a known limitation of the single-cell method utilized.[40] The majority of immune cells detected in the single-cell analysis were T cells and myeloid cells where proportions for each immune cell subtype differed between patients (Figures 1F, S1C, and S1D). In addition, we detected T cells and macrophages for most of the samples we performed immunohistochemistry on, and immune cell infiltration overall was between 5% and 35% of total cells in the tumor (Figures 1G and S1E). Next, we created a combined dataset containing data from previous NB scRNA-seq studies[25–27] and we determined the fraction of immune cells in low-, intermediate-, and high-risk disease. We detected no differences in the major immune cell populations comparing the risk groups (Figure S1F). Survival analysis on bulk RNA sequencing data for 498 samples, based on gene expression signatures derived from our scRNA-seq (Table S3), showed that high expression of immune-related genes was significantly correlated with improved survival in human NB (Figure 1H).

Infiltration of myeloid cells with distinct cell states detected in NB

Myeloid cell infiltration has been described for multiple cancers and shown to support tumor growth.[18,41,42] In NB, discrepancies between studies and a lack of study into myeloid cell heterogeneity in NB prompted us to analyze these cells in detail.

We have used *in silico* subcluster analysis[32] focusing on the myeloid cells present within NB tissues obtained by our lab to identify nine distinct subpopulations. These we annotated as Mono-1/2, CLEC9A+ myeloid dendritic cells (mDCs), CD1C+ mDCs, mature-LAMP3+ mDCs, and Macro-1, 2, 3, and 4, based on their expression of key marker genes for respective cell lineages (Figures 2A–2C). Multiplex immunohistochemistry, on patient samples from the single-cell cohort (Table S1), was used for the detection of antigen-presenting myeloid cells (CD11c+, five out of five patients, and HLA-DR+, in three of three patients) (Figures S2A and S2B). To characterize the cell state of the myeloid cells, we curated a gene signature score based on existing scRNA-seq studies describing previously characterized tumor-derived monocyte and macrophage cell states (Table S4). Our analysis revealed a significantly higher monocyte score in both Mono-1 and Mono-2 compared with the other myeloid populations (Figures S2C and S2D), substantiating that these cells are monocytes. Macro-2 and Macro-3 also had a high monocyte score (Figures S2C and S2D). Furthermore, Macro-1 showed the highest macrophage cell identity score followed by the three other macrophage populations when compared with the monocytes (Figures S2E and S2F), substantiating their macrophage cell state.

Focusing on Mono-1/2 subclusters, we annotated these clusters based on the expression of the key monocyte marker genes *CLEC7A*, *NLRP3*, and *BST1*.[43] Both monocyte clusters expressed the pro-inflammatory cytokine *IL1B*, the monocyte genes *TIMP1*, *CD44*, and *G0S2*, and several inflammatory-related *S100A* genes (Figures 2B and 2C). Mono-1 appeared to consist of two subpopulations within: a small population of non-classical monocytes with expression of *FCGR3A*, *FCGR3B*, and *IFITM3*, and a larger population of cells that exhibited specific expression of *TIMP1*, *S100A4*, *S100A12*, and *CD55* (Figures 2B and 2C). Mono-2 had a mixed gene expression pattern with similarities to both monocytes and macrophages. This population might be differentiating into Macro-2 since part of the population displayed expression of the

(G) Quantification of percentage of cells from (E) (n = 43).
(H) Survival data on bulk RNA-seq data. A gene signature derived from scRNA-seq high was considered the top 25% highest expression of the signature genes, whereas low is the lowest 25% expression of the signature genes (STAR Methods).

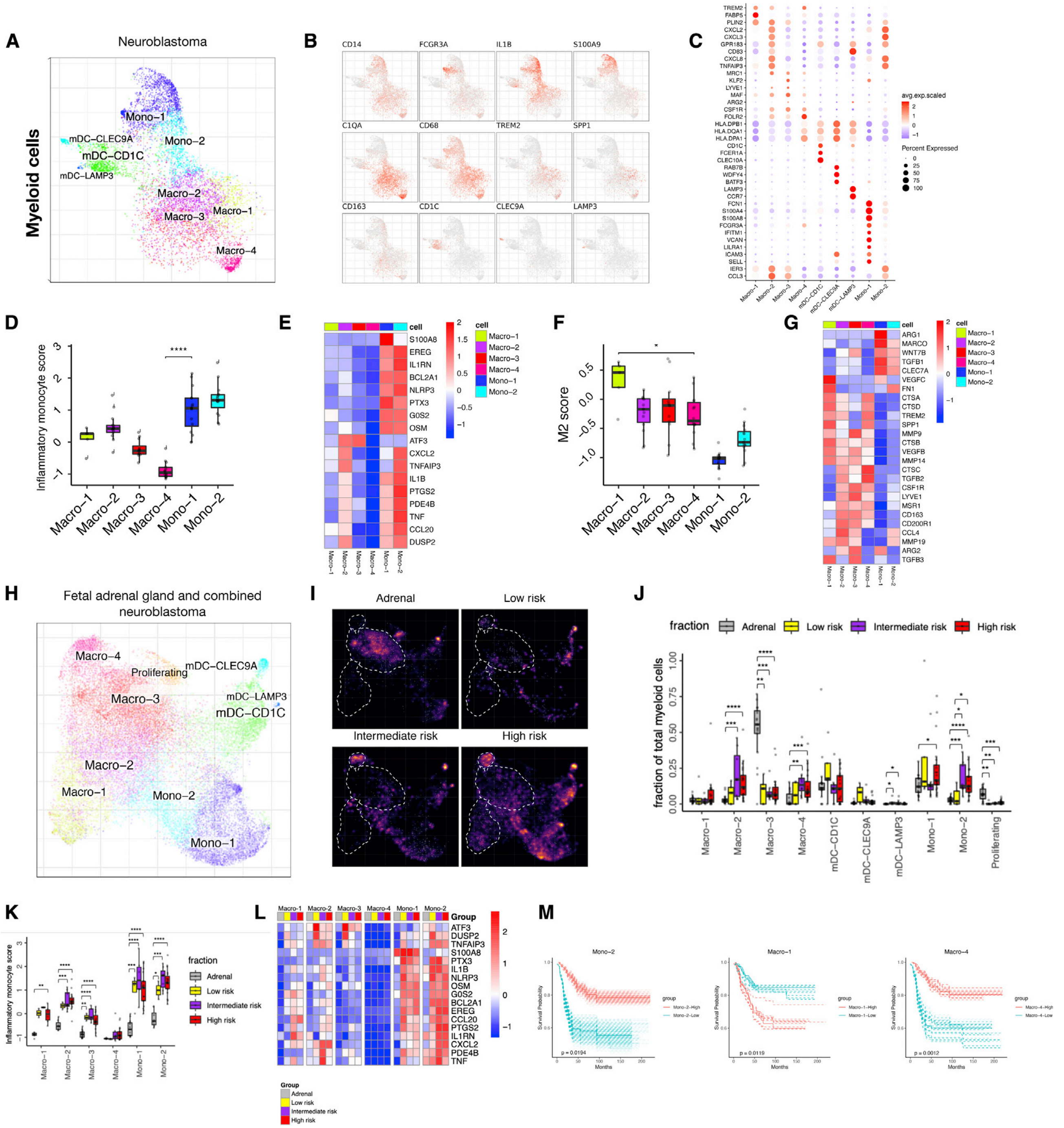

Figure 2. Myeloid cell infiltration with distinct cell states detected in NB

(A–C) (A) Subcluster view of the myeloid cells as shown on a myeloid-specific joint embedding. Key marker gene expression shown in feature plots (B) and in a dotplot (C) for the different subpopulations of myeloid cells.

(D) Average expression of inflammatory monocyte score in different myeloid subpopulations (n = 16).

(E) Heatmap showing average expression of select genes from different categories (rows) across different cell populations.

(F and G) Similar to (Dand E), showing M2 score (n = 16) and representative M2 signature gene expression.

(H) UMAP showing combined myeloid cell integration (CONOS) including fetal adrenal and public NB single-cell data.

(I) Density plot comparing myeloid cells in fetal adrenal gland myeloid cells, and low-, intermediate-, and high-risk NB. Brighter color corresponds to a denser region.

(J) Cell fractions of different myeloid populations in fetal adrenal gland (n = 16), and low- (n = 5), intermediate- (n = 8), and high-risk (n = 21) disease.

(legend continued on next page)

macrophage marker genes *CD68* and *APOE* (Figures 2B and 2C).[35] To provide a possible function of Mono-1/2, we applied a functional gene signature score analysis focusing on the inflammatory activity of monocytes, and compared them to macrophages (Table S4). Our analysis showed that Mono-1/2 have a significantly higher inflammatory monocyte score compared with macrophage populations and are therefore most likely pro-inflammatory monocytes (i.e., TIMs) (Figures 2D and 2E) that we previously characterized in human prostate cancer bone metastases.[32]

Further investigation of the macrophage populations showed that the macrophage clusters had high expression of the classic macrophage marker gene *CD68* (Figure 2B).[35] Furthermore, Macro-1 displayed a population-specific gene expression pattern of tumor-associated macrophage markers, such as *TREM2*, *FABP5*, and *FABP4* (Figures 2B and 2C).[44–46] Macro-2 expressed high levels of neutrophil-attracting chemokines, including *CXCL2*, *CXCL3*, and *CXCL8*.[47] In addition, this population exhibited high expression of *GPR183*, *DUSP2*, *CD83*, and HLA class II genes (Figures 2B and 2C). Macro-3 displayed expression of other macrophage genes, such as *LYVE1*, *CSF1R*, and *MRC1*,[20,45,48] and Macro-4 had the highest level of HLA class II genes (Figures 2B and 2C). To demonstrate the possible functional properties of the different macrophage populations, we ran analysis of M1 (i.e., immune active) and M2 (i.e., immunosuppressive) signature score (Table S4), which revealed that Macro-1 had a significantly higher M2 score than the other macrophage populations, and those in turn had a higher M2 score compared with the monocytes (Figures 2F and 2G). Among Macro-1 to 4, Macro-2 had the highest M1 signature score (Figures S2G and S2H).

We next examined the myeloid dendritic cell population that branched into three different subpopulations, including CLEC9A+ cells, CD1C+ cells, and mature-LAMP3+ cells. CLEC9A+ mDCs expressed high levels of *CLEC9A*, *RAB7B*, *BATF3*, *WDFY4*, and *CADM1*, which phenotypically corresponded to DC1 cells (Figures 2A–2C).[43] CD1C+ mDCs were identified by the high expression of *CD1C*, *CLEC10A*, and *FCER1A*, but also exhibited high expression of HLA class II antigen-presenting genes, and therefore had a similar phenotype to previously described DC2.[43] Mature-LAMP3+ mDCs showed high expression of *CCR7* and *CD83* (Figures 2B and 2C).

To increase the power of our analysis, but also to compare our findings with corresponding normal tissue for NB, we combined our dataset with three recently published scRNA-seq studies (including a recent study[25]) of normal fetal adrenal tissue (i.e., where NB is believed to originate) and NB tumors (see section Data and code availability in STAR Methods).[25–27] Here, we observe that the fetal adrenal data mainly contained myeloid cells (Figures S2I and S2J). In the combined dataset, we detected the same myeloid subpopulations as in our dataset, validating our findings (Figures 2H and S2K), where the annotations were based on key marker genes (Figure S2L). To determine the role of specific myeloid populations on disease risk stratification, we compared the fraction of cells and the different functional gene signature scores from the combined dataset[25–27] in low-, intermediate-, and high-risk NB compared with healthy fetal adrenal myeloid cells. Our analysis revealed that the main myeloid population found in fetal adrenal tissue is Macro-3. Also, these cells showed significantly lower cell fractions in NB patients, while Macro-4 demonstrated an increase in the intermediate- and high-risk NB (Figures 2I and 2J). In addition, Macro-3/4 were close together in the embedding (Figure 2H). Looking into cell state, the different gene signature scores showed a significantly increased M1 score for four out of six populations, and a significantly decreased M2 score with higher disease risk in Macro-3 and Mono-2 (Figures S2M and S2N). Surprisingly, we discovered a significant increase of inflammatory monocyte score in all populations except Macro-4, indicating that the presence of tumor cells strongly initiates inflammatory gene expression in these cells (Figure 2K). Focusing on Mono-1 and Mono-2 in this scoring analysis, we also detected a difference in gene expression comparing fetal adrenal and NB cells (Figure 2L).

Finally, to evaluate for potential prognostic value of different myeloid cell populations, we ran survival analysis on bulk RNA sequencing data based on key marker gene expression from our scRNA-seq dataset (Figures 2C; Table S5). Interestingly, Mono-2 and Macro-4 significantly correlated with improved survival, whereas Macro-1 was significantly correlated with decreased survival (Figure 2M). Signatures based on CLEC9A+ and CD1C+ dendritic cell populations were associated with improved survival, whereas the other monocyte and macrophage populations and mature mDCs did not show an association with survival (Figure S2O). Since NB is a clinically heterogeneous disease, we split the bulk RNA sequencing data to determine survival into low- and high-risk cases, and into non-MYCN-amplified and MYCN-amplified cases. Next, we applied the gene scores for the different myeloid cell populations on the split data and created a summary figure. There, we detected a significant correlation with improved survival for CD1C mDCs in the low-risk group, but there were no differences for the other groups (Figure S2P).

B and NK cell heterogeneity found in NB

We next focused our analysis on the B and NK cells. Subcluster analysis of the B cells in the combined dataset revealed the presence of four subpopulations in NB tissues (Figures 3A–3C). B cell infiltration was confirmed by multiplex immunohistochemistry on patient samples from the single-cell cohort (Table S1, Figures S3A and S3B). Active B cells expressed B cell marker genes, such as *IGHD*, *IGHM*, *CD69*, and *CD83*,[49] and plasma cells expressed high levels of *MZB1* and *JCHAIN* (typical B cell plasma marker genes) (Figures 3B and 3C).[50,51] In addition, we detected memory B cells that were annotated based on the expression of *CD27*, *CLECL1*, and *ZBTB32* (Figures 3B and 3C).[52] We also

(K) Inflammatory monocyte score for combined dataset comparing fetal adrenal gland (n = 16), and low- (n = 5), intermediate- (n = 8), and high-risk (n = 21) NB for different myeloid subpopulations. Statistical significance was assessed by Wilcoxon rank-sum test for (D, F, J, and K); *p < 0.05, **p < 0.01, ***p < 0.001, ****p < 0.0001.

(L) Heatmap showing average expression of select genes from different categories (rows) across different cell populations (top color bar, colors matching) (K).

(M) Similar to Figure 1E, survival curves for Mono-2, Macro-1, and Macro-4.

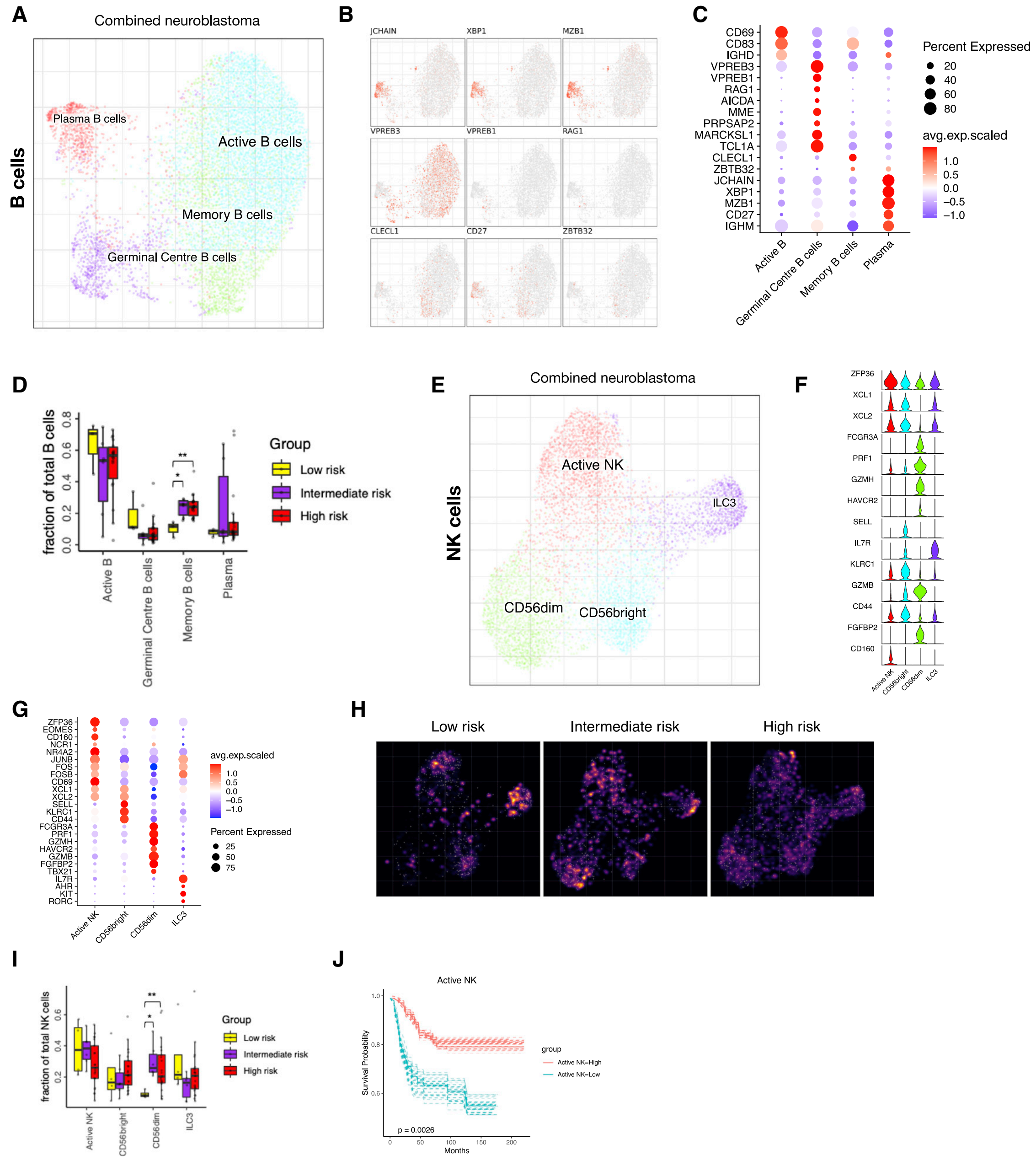

Figure 3. B and NK cell heterogeneity and infiltration in NB tumors

(A–C) (A) Detailed annotation of the B cells subpopulations is shown on a B cell-specific joint embedding combining multiple NB datasets. Key marker gene expression shown in feature plots (B) and in a dotplot (C) for the different subpopulations of B cells.

(D) The fraction of B cell subtypes comparing low- (n = 3), intermediate- (n = 6), and high-risk (n = 18) NB. Wilcoxon rank-sum test was used for statistical analysis; *p < 0.05, **p < 0.01.

(E–G) (E) Detailed annotation of NK cell subpopulations shown on NK cell-specific embedding from the combined dataset. Key marker gene expression shown in a violin plot (F) and in a dotplot (G) for the different subpopulations of NK cells.

(H) Density plot for NK cell-specific embedding showing the number of cells in low-, intermediate-, and high-risk NB. Brighter color corresponds to a denser region.

(I) The fraction of cells in the different NK cell populations comparing low- (n = 4), intermediate- (n = 6), and high-risk (n = 21) NB. Wilcoxon rank-sum test was used for statistical analysis; *p < 0.05, **p < 0.01.

(J) Survival curve for active NK cells.

CellPress
OPEN ACCESS

discovered the presence of germinal center (GC) B cells. GC B cells expressed high levels of VDJ recombination genes, including *VPREB3*, *VPREB1*, and *RAG1*.[53] Furthermore, this GC cluster exhibited expression of previously described GC-associated genes, such as *AICDA*, *MME*, *PRPSAP2*, and *MARCKSL1* (Figures 3B and 3C).[50,54,55] Using immunohistochemistry, we detected a B cell aggregate combined with antigen-presenting cells in one of our five patient samples showing the presence of tertiary lymphoid structures (Figures S3A and S3B).

Furthermore, B cells were barely detected in the human fetal adrenal glands and therefore excluded from the joint analysis. Comparing low-, intermediate-, and high-risk disease, we detected a significant increase in memory B cells and a decrease in GC B cells in intermediate- and high-risk disease compared with low-risk tumors (Figures 3D and S3C). In addition, we determined the prognostic value of the different B cell subtypes in a similar manner as performed in Figure 1H and for the myeloid subpopulations (genes can be found in Figure 3C and Table S5), where we observed a trend in the presence of plasma cells and tertiary lymphoid structure (TLS) signature score with survival; however, the results were not statistically significant (Figures S3D and S3E). Finally, in a similar approach as for the myeloid cells, we created a survival summary figure showing the different clinical conditions, where we detected no significant correlation with survival (Figure S3F).

NK cells have potent tumor-killing properties, and NK cell infiltration has been demonstrated as a good prognostic marker in different cancers.[56,57] In the combined NB dataset, subcluster analysis of the innate lymphoid cells (ILCs) identified four subclusters (Figure 3E). No NK cells were detected in the fetal adrenal glands (data not shown). The NK-active cell cluster was annotated based on the expression of immediate-early genes, such as *ZPF36*, *NR4A2*, *JUNB*, *FOS*, *FOSB*, and *CD69*.[39] Classical NK cell subsets $CD56_{bright}$ and $CD56_{dim}$ were detected (Figures 3F and 3G).[39,58] In addition, an ILC3 cell cluster was annotated based on their lack of expression of classical NK cell marker genes *TBX21* and *EOMES* and the high expression of *AHR*, *KIT*, *IL7R*, and *RORC* (Figures 3F and 3G).[59]

The potently cytotoxic $CD56_{dim}$ NK cells (Figure S3G) were found significantly enriched in cell number in intermediate- and high-risk disease (Figures 3H, 3I, and S3H). However, their presence in NB did not correlate with improved survival (Figure S3I). On the other hand, active NK cells were significantly correlated with improved survival (Figure 3J). No significant differences were detected when the data were split by risk group and MYCN status (Figure S3J). NK cell survival analysis was done as described above using key marker gene expression (Figure 3G; Table S5).

Distinct subtypes of T cell infiltrates correlate with improved NB survival

Various subpopulations of T cells have been suggested to play a critical role in cancer development and progression, which has prompted the identification of new immuno-therapeutic approaches based on modulating T cell function and dysfunction in cancer.[60–63] In our NB dataset, subcluster analysis revealed ten distinct subclusters of T cells, including two $CD4^+$ clusters (Tregs [regulatory T cells] and Th17), four cytotoxic T lymphocyte (CTL) $CD8^+$ clusters (CTL-1/4), a naive T cell cluster containing cells with *CD4* expression and cells with *CD8A/B* expression, a proliferating T cell cluster, and an NKT cell cluster (Figures 4A–4C). Infiltration of the different T cell subtypes was validated by multiplex immunohistochemistry (patient information in Table S1) and single staining immunohistochemistry (patient information in Table S2) (Figures S4A–S4D). Single-cell transcriptomics and immunohistochemistry both showed the presence of Th17 cells (*RORC*, *CCL20*, *CCR6*, and, less prominently, *IL17A*).[64] However, other cell types, such as ILC3[59] and γδT cells,[65] can also produce IL-17, where the latter promote tumor cell proliferation and migration in NB cell lines.[65]

Tregs were identified based on *FOXP3* and *IL2RA* expression, two key Treg marker genes[66] (Figures 4B and 4C), and infiltration of Tregs was validated by FOXP3 staining (Figures S4C and S4D). Because of their tumor-promoting role in adult cancer, we sought to evaluate the activity of Tregs in NB based on a gene signature score curated from literature (Table S4). Treg activity was evaluated in the combined dataset, where we detected no difference in Treg activity score comparing low-, intermediate-, and high-risk disease (Figures S4F and S4G). Surprisingly, the number of Tregs showed a significant decrease from intermediate-to high-risk disease (Figure 4D). In addition, Tregs did not correlate with survival (Figure S4H). Due to the low number of T cells in the normal fetal adrenal data this was not included in the combined data analysis.

Next, we sought to delineate the CD8+ T cell compartment, which revealed four different subtypes of cytotoxic T lymphocytes where CTL-1 cells expressed high levels of *PRF1* and *GZMB* (Figures 4A–4C). The expression of granzyme-B and perforin-1 showed high cytotoxic properties for CTL-1. CTL-4 expressed high levels of *TNF*, *CD69*, and *IFNG*. Cytotoxic T cells upregulate the expression of *CD69* and *IFNG* upon TCR stimulation,[67,68] showing that CTL-4 are activated T cells (Figure 4B). To further investigate the functional properties of the CTL populations in NB, we took the combined dataset (Figure S4E) and performed cytotoxicity and exhaustion signature score analysis comparing low-, intermediate-, and high-risk disease (Table S4). Interestingly, no differences were found for both cytotoxicity and exhaustion score for the different CTL populations in NB (Figures S4I and S4J).

Since different T cell subtypes have rendered diverse results in prognostic value in different NB studies,[5,15,69,70] we ran survival analysis for the different T cell subtypes based on their marker genes (Figure 4C; Table S5). Including all data, CTL-1, CTL-3, CTL-4, naive T cells, and Th17 signatures were significantly correlated with improved survival (Figure 4E), whereas proliferating T cells, CTL-2, and NKT cell signatures did not (Figure S4K). When we split the data into risk group and divided by MYCN status as described for other cell types above, we detected no significant differences (Figure S4L).

Ligand-receptor interaction analysis reveals a highly complex interactive network of immune cells with tumor and stroma

To investigate potential interactions and molecular crosstalk between cells located within the NB microenvironment,

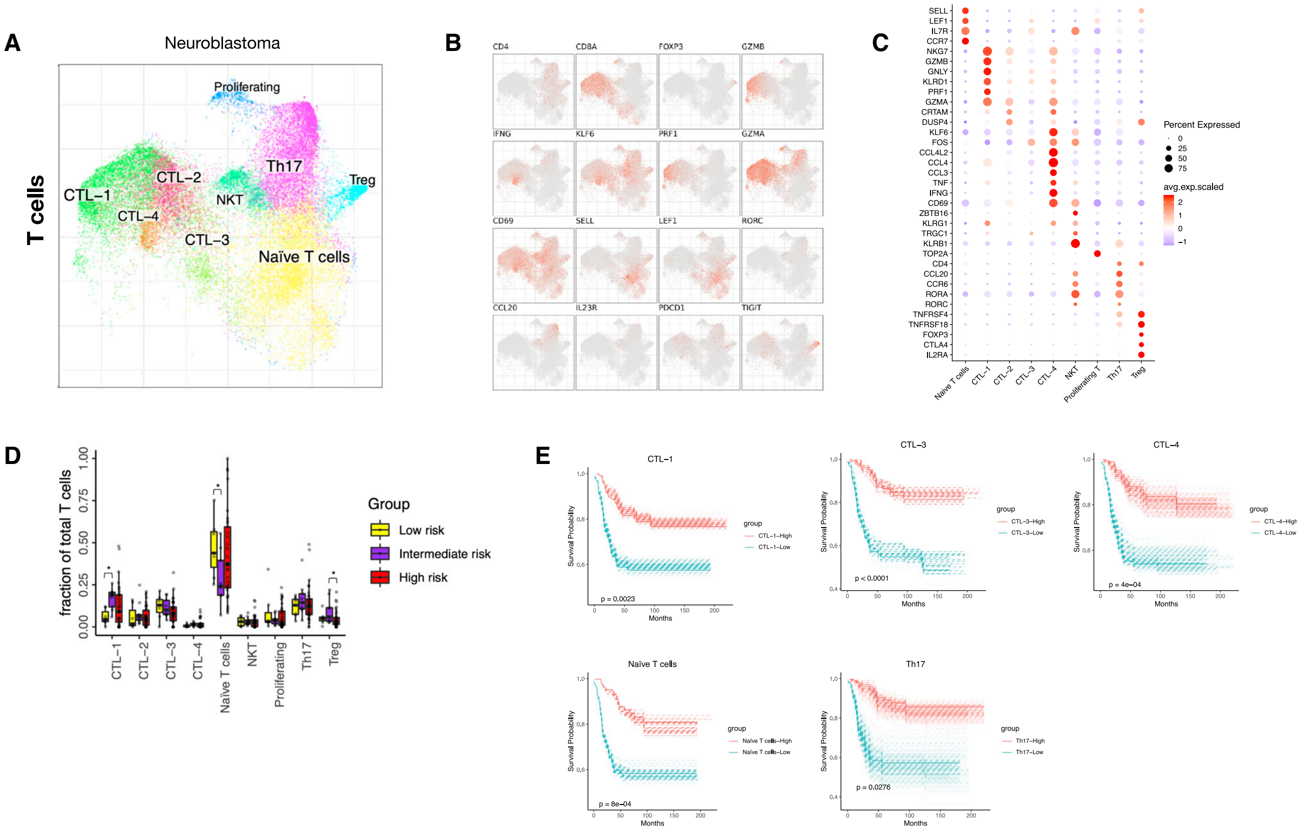

Figure 4. Distinct subtypes of T cell infiltrates correlate with improved NB survival

(A–C) (A) Detailed annotation of the T cell subpopulations shown on a T cell-specific joint embedding, together with marker genes (B and C).

(D) The fraction of T cell subtypes detected in low- (n = 4), intermediate- (n = 9), and high-risk (n = 23) NB. Wilcoxon rank-sum test was used for statistical analysis; *p < 0.05.

(E) Survival curves for CTL-1, -3, -4, naive T cells, and Th17.

we performed receptor-ligand interaction analysis.[71] To find interactions that may be targetable, we used significant correlation to survival as a cutoff. In addition, we set a cutoff for specific expression of both ligand and receptor in a specific cellular subtype. Our analysis revealed that the tumor microenvironment of NB consists of a highly complex interacting cellular network (Figure 5A). Most of the interactions involving tumor cells occurred with the different stroma cell populations, but there were also noticeable interactions within tumor/stroma cells and the immune cells (Figures S5A and S5B). Importantly, we detected a high number of potential interactions between stroma and immune cells, involving CTL1, mesenchymal, and endothelial populations (Figure 5B). Next, to investigate specific cellular interactions in our dataset, we generated a list of the top ligand-receptor interactions for tumor/stroma cells versus immune cells (Figure 5C), tumor/stroma cells versus tumor/stroma cells (Figure S5A), and immune cells versus immune cells (Figure S5B). Our analysis detected genes well studied in the NB field in addition to other interesting interactions. The genes included the presence of NOTCH pathway ligand and receptor genes known to play a role in NB tumor cell differentiation[72,73] and the expression of NRTK1 specifically in tumor cells, which, when activated, is described to cause neuronal differentiation and cell-cycle arrest in NB.[74] Subsequently three particularly interesting interactions (based on highest expression) drew our attention due to their relation to already described importance in cancer and correlation to survival. These may therefore be used as possible modulatory targets for NB treatment: semaphorin 6D (*SEMA6D*)/triggering receptor expressed on myeloid cells 2 (*TREM2*), galectin-9 (*LGALS9*)/hepatitis A virus cellular receptor 2 (*HAVCR2*), and cluster of differentiation 24 (*CD24*)/sialic acid binding Ig-like lectin 10 (*SIGLEC10*) (Figure 5C). Survival analysis of the indicated interactions showed that high expression of *SEMA6D-TREM2* was significantly correlated with decreased survival (Figure 5D), suggesting this interaction as a possible target in NB. On the contrary, high *LGALS9-HAVCR2* expression was positively correlated with survival (Figure 5E). In a similar manner, high *CD24-SIGLEC10* expression was significantly correlated with improved survival (Figure 5F). This suggests that *LGALS9-HAVCR2* and *CD24-SIGLEC10* might possibly be used in a different manner than the classical "inhibition to unleash activity" strategy.[75] Understanding and exploiting these interactions will require additional studies.

CellPress
OPEN ACCESS

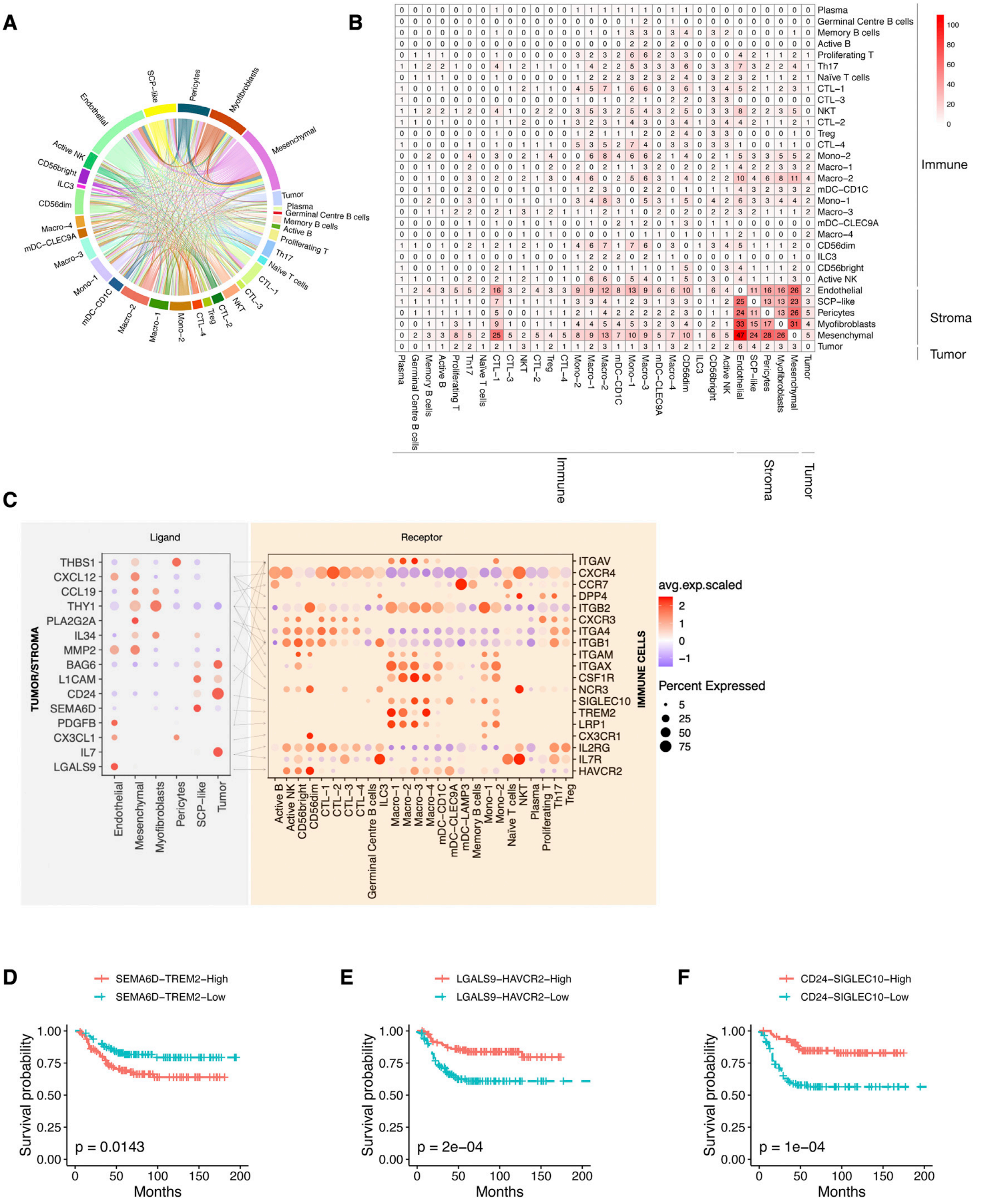

Figure 5. Tumor-immune cell ligand-receptor interaction analysis reveals several interactions for future studies

(A) Interaction map with ligand-receptor interactions within the NB tumor microenvironment (n = 19).

(B) Table displaying the number of interactions (n = 1,973) between the different subpopulations present in human NB tumors that are significantly correlated with patient survival and specifically expressed in a subtype of cells.

(C) Heatmap showing expression of ligand (tumor/stroma) and receptor (immune) pairs in different tumor/stroma and immune subsets. Dot size indicates expression ratio, color represents average gene expression. Significance of ligand-receptor pair is determined by permutation test, correlation to survival and specific cellular expression (see STAR Methods).

(D) Survival curve for *SEMA6D-TREM2* expression.

(E) Survival curve for *LGALS9-HAVCR2* expression.

(F) Survival curve for *CD24-SIGLEC10* expression.

DISCUSSION

Here, we created a comprehensive cellular and molecular map of immune cells present within the NB microenvironment and provided a resource for future research into the development of novel therapeutics. We used a combination of single-cell transcriptomics, multiplex immunohistochemistry, and *in silico* analysis. The cell atlas shows 27 different immune cell subtypes that create a highly unique interactive network of cellular and molecular interactions within immune populations and with tumor and stroma cell populations in NB.

It is important to emphasize here that analyzing scRNA-seq from different studies where different tissue dissociation protocols were used might introduce bias in terms of cell fraction changes. We were therefore cautious when interpreting cell fraction differences since distortions from protocols are still likely to be pronounced. Instead, we focused our analyses on cell-state changes that should provide a more accurate analysis.[32] In addition, it is important to note that 7 out of the 17 patients included in our study were pre-treated and/or treated with systemic therapy at the time of tissue harvest. Before tissue harvest, patients had at least 2 weeks of wash out after chemotherapy and none of the patients were treated with immunotherapy. Recently, the first study trying to understand the influence of therapy on immune cells in the blood of high-risk NB patients was conducted. Interestingly, in this study, large variations between patients existed and immune cell phenotypes related to both tumor suppression and support were detected.[76] The influence of different cancer therapies on immune cells in the broader cancer perspective has not been defined yet, with chemotherapy having both immune enhancing and inhibiting properties.[77] Furthermore, interpretation of survival analysis can be difficult since the outcome is highly dependent on the type of analysis, the number of patient samples, the distribution of outcome, which biomarkers are analyzed, and possible additional factors. For several cell types, we detected a significant correlation with improved survival overall. However, when splitting the samples into different risk group and MYCN status the differences disappear. This may be due to the fact that low-risk patients do well overall; therefore, the number of high-risk patients goes down and it is difficult to detect differences. Other factors than tumor biology (like treatment) may influence outcome within the different subgroups (risk group/MYCN status); however, the limited number of patients did not allow us to study those. Nevertheless, the overall analysis and interpretation we have done is important for clinical purposes.

Myeloid cell infiltration has been described for multiple cancers and shown to support tumor growth.[18,41,42] In NB, CSF1R+ myeloid cells have been shown to predict poor clinical outcome[20]; however, other studies have indicated myeloid cells to be correlated with improved survival.[78] The different myeloid cell subtypes in our study show a gene expression pattern indicating ongoing plasticity in both monocytes and macrophages, and we detected cells with pro-inflammatory phenotypes, which is in contrast to previous studies regarding myeloid cells in NB.[20,78] Pro-inflammatory immune cells and inflammation can promote tumor growth and cause metastatic spread in cancer.[79] However, our data suggest that, in NB, a pro-inflammatory state is a good prognostic factor due to the positive correlation between different myeloid cell populations with pro-inflammatory cell states and improved survival. The different myeloid populations may still be exploited in the pursuit for novel therapeutics. Several interactions exist between tumor/stroma and myeloid cells, which could be exploited as therapeutic targets to recruit the good prognostic monocyte and macrophage players into the NB tumor. For this, we suggest further in-depth studies that will hopefully demonstrate their specific function and the possible use of these interactions in therapeutic strategies.

B cells have gained significantly more attention in the cancer field. Three studies demonstrated that B cells and the presence of TLSs in adult tumors correlated with improved response to immunotherapy in melanoma and sarcoma.[51,80,81] In human NB, a paper on the interaction between dendritic cells and NK cells detected the presence of TLSs.[14] Since TLSs are dynamic structures containing different immune cell types, they may be highly interesting for targeting or supporting therapy.

T cells, mainly CTLs, have been extensively studied in cancer. CTLs can play an important role in cancer eradication and have therefore been the main targets of currently used immunotherapy such as PD-L1 checkpoint inhibitors.[82] In our study, we showed that different CTLs are an important part of the interacting NB microenvironment and that several CTL subtypes correlated with improved survival. Importantly, we did not detect differences in exhaustion score across NB risk groups, implying limited phenotypic changes over the course of disease. Moreover and surprisingly, Tregs were not correlated with survival, a finding that is in contrast with Treg suppression and exhaustion of effector cells observed in adult cancer.[83] This indicates that the NB tumor microenvironment differs from adult cancer. Another possible explanation might be a result of dilution in bulk RNA sequencing from survival datasets to the low number of Tregs in the tumor microenvironment.

Our receptor-ligand interaction analysis between tumor/stroma and immune cells revealed several genes that have already been correlated with NB growth and differentiation,[72–74,84] as well as the discovery of important cell-cell interactions mediating immune suppression in other cancer types, such as the CCL20-CCR6 axis in prostate cancer bone metastasis[32] and the CD161-CLEC2D axis in glioma.[85] This provides a basis for the additional genes detected in our analysis to have a potential therapeutic impact. For instance, SEMA6D was expressed mainly on SCP-like cells and functions as an inhibitory ligand upon binding to Plexin A1, which forms a receptor complex with TREM2, a marker for TAMs.[86] TREM2 deficiency has been shown to attenuate tumor growth, and combining TREM2 deficiency with PD-1 antibodies caused tumors to completely regress in a sarcoma mouse model.[87] Since we detected that *SEMA6D-TREM2* expression is significantly correlated with decreased survival suggesting, we suggest this interaction as a potential therapeutic target. Furthermore, we detected elevated *LGALS9* expression in endothelial cells that interacts with *HAVCR2* (encodes TIM-3) on NK cells. TIM-3 present on NK cells serves as a maturation marker and functions as an inhibitory receptor, while TIM-3 induced inhibition can be overcome by

cytokine stimulation.[88] The function of TIM-3 in NK cells upon galectin-9 binding and immune cues differs depending on the cues and the endurance of the input, rendering TIM-3 function in NK cells in different cancers controversial. It has been suggested that the initial upregulation of TIM-3 enhances cytotoxicity and that chronic overexpression or dysregulation leads to exhaustion.[89] However, the precise role of TIM-3 in NK cell function in NB cannot be determined based on our analysis, and therefore warrants further investigation. Finally, the surface receptor *CD24* was exclusively expressed on tumor cells and interacts with *SIGLEC10* on macrophages in the immune cells. CD24 is a "don't eat me" signal, which conveys an inhibitory response in macrophages when binding to SIGLEC10 preventing these macrophages from exerting phagocytosis. The presence of CD24 has been detected on several solid tumors and its importance in suppressing phagocytosis has recently been demonstrated in ovarian and breast cancer. In addition, it is suggested that CD24/SIGLEC10 might be a target for cancer immunotherapy.[90] Hence, monoclonal anti-CD24 antibodies could be investigated as a potential therapy in NB.

Immune cells and immunotherapy in NB are an active field of research. Despite lack of MHC class I and high mutational load in NB tumors, major steps toward improved therapy are being made. Two recent studies demonstrated the potential of immunotherapy in NB by improving current anti-GD2 to reduce pain and by creating novel chimeric antigen receptors(CARs) on T cells toward a peptide sequence derived from the master transcriptional regulator in NB PHOX2B.[23,91] Our study provides a comprehensive resource for possible ways the tumor microenvironment could be exploited to further enhance immunotherapy in NB.

In conclusion, our study suggests that the immunological landscape in the NB microenvironment appears and most probably functions differently from the well-characterized adult cancer tumor microenvironment. Since NB is known to be initiated early during embryonal development,[1,31] it may be possible that tumor development occurred alongside immune cell development and therefore other mechanisms are at play within the NB microenvironment. We believe that our findings will pave the way to a better understanding of the role of the immune system in NB. It is important to emphasize that future studies are needed to understand the functional properties of each immune cell subtype and the suggested cell-cell interactions between the different cell types that our study has identified. Combined, we hope that these biological insights will help to tailor new and improved therapeutics that instruct the immune system against NB.

Limitations of the study

While the presented analysis provides a good representation of immune cell heterogeneity in NB in general, a number of potential limitations should be noted. First, our dissociation protocol used for preparing single-cell suspensions is known to enrich for immune cells.[32] We, therefore, observe a higher frequency of immune cells relative to tumor and stromal cells in our dataset compared with public datasets and were cautious about interpreting frequency data outside of the data obtained using the same protocol. This enrichment, however, gave us the opportunity to study immune cell granularity in NB in great detail. Second, we have performed interpretation of functionality of certain immune cell types based on gene expression data. Protein validation and additional functional studies will have to provide additional evidence for these interpretations. Finally, creation and interpretation of survival curves based on gene expression can be challenging. NB is a highly heterogeneous disease in which genetics, risk group, age, and treatment can strongly influence outcome, and gene expression values are not the optimal choice for assessing survival. Despite the above-mentioned reasons, we managed to detect significant differences, which we believe provide important clinical insights into the influence of immune cells on NB survival.

STAR★METHODS

Detailed methods are provided in the online version of this paper and include the following:

- KEY RESOURCES TABLE
- RESOURCE AVAILABILITY
  - Lead contact
  - Material availability
- EXPERIMENTAL MODEL AND SUBJECT DETAILS
  - Patient material and collection of tumor specimens
  - Tumor material used for immunohistochemistry
  - Ethical approval for material used in immunohistochemistry
- METHOD DETAILS
  - Dissociation of NB into single cells
  - FACS enrichment of viable NB cells
  - Single cell library preparation and sequencing
  - Sectioning of NB tumors
  - Highly multiplexed immunofluorescence
  - Immunohistochemistry
  - Infiltration density of immune cells in human NB
- QUANTIFICATION AND STATISTICAL ANALYSIS
  - Pre-processing of scRNA-seq data
  - ScRNA-seq analysis
  - Analysis of cell composition shift
  - Calculation of gene set signature scores
  - Survival analysis
  - Identification of significant ligand-receptor pairs
  - Statistics
  - Data and code availability

SUPPLEMENTAL INFORMATION

Supplemental information can be found online at https://doi.org/10.1016/j.xcrm.2022.100657.

ACKNOWLEDGMENTS

We thank Conny Tümmler, Bethel Embaie, Ioanna Tsea, and Baldur Sveinbjornsson for reviewing and commenting on the manuscript. B.M.V., P.K., J.I.J., and N.B. were funded by the Swedish Childhood Cancer Foundation, the Swedish Cancer Society, and the Radiumhemmet Research Foundations. P.K. and J.I.J. were funded by the Swedish Childhood Cancer Foundation, the Swedish Cancer Society, the Radiumhemmet Research Foundations, and the

Swedish Foundation for Strategic Research. T.K.O., N.B., and P.K. were financially supported by the Knut and Alice Wallenberg Foundation as part of the National Bioinformatics Infrastructure Sweden at SciLifeLab. P.V.K. was founded by the NIH R01HL131768 grant from NHLBI. C.H. was funded by the Swedish Childhood Cancer Foundation, the Swedish Society of Medical Research, the Gunnar-Nilsson Cancer Foundation, and governmental funding of clinical research within the National Health Services (ALF). S.S.F. was funded by the Swedish Childhood Cancer Foundation. We thank Caroline Jansson for performing immunohistochemistry staining and Sofie Mattson for quantifying immune cells in NB tumors. We acknowledge the Spatial Proteomics Facility at Scilifelab, funded by Scilifelab and the National Microscopy Infrastructure, NMI (VR-RFI 2019-00217), for providing highly multiplexed immunofluorescence experiments with the CODEX platform.

AUTHOR CONTRIBUTIONS

Conceptualization, B.M.V. and N.B.; investigation, B.M.V., S.M., and N.B.; collection of samples, T.K.O. and P.K.; validation, B.M.V., S.S.F., A.V., A.L., D.G., and C.H.; computational investigation and analysis, B.M.V., T.K.O., S.M., P.V.K., and N.B.; writing – original draft, B.M.V. and N.B.; writing – review & editing, B.M.V., P.V.K., C.H., J.I.J., P.K., and N.B.; funding acquisition, resources, and supervision, N.B.

DECLARATION OF INTERESTS

P.V.K. serves on the Scientific Advisory Board to Celsius Therapeutics and Biomage and also consults for Altos Labs.

Received: November 7, 2021
Revised: January 26, 2022
Accepted: May 17, 2022
Published: June 9, 2022

REFERENCES

1. Johnsen, J.I., Dyberg, C., and Wickström, M. (2019). Neuroblastoma—a neural crest derived embryonal malignancy. Front. Mol. Neurosci. *12*, 1–11. https://doi.org/10.3389/fnmol.2019.00009.
2. Pinto, N.R., Applebaum, M.A., Volchenboum, S.L., Matthay, K.K., London, W.B., Ambros, P.F., Nakagawara, A., Berthold, F., Schleiermacher, G., Park, J.R., et al. (2015). Advances in risk classification and treatment strategies for neuroblastoma. J. Clin. Oncol. *33*, 3008–3017. https://doi.org/10.1200/jco.2014.59.4648.
3. Gajewski, T.F., Schreiber, H., and Fu, Y.X. (2013). Innate and adaptive immune cells in the tumor microenvironment. Nat. Immunol. *14*, 1014–1022. https://doi.org/10.1038/ni.2703.
4. Vinay, D.S., Ryan, E.P., Pawelec, G., Talib, W.H., Stagg, J., Elkord, E., Lichtor, T., Decker, W.K., Whelan, R.L., Kumara, H.M.C.S., et al. (2015). Immune evasion in cancer: mechanistic basis and therapeutic strategies. Semin. Cancer Biol. *35*, S185–S198. https://doi.org/10.1016/j.semcancer.2015.03.004.
5. Mina, M., Boldrini, R., Citti, A., Romania, P., D'Alicandro, V., De Ioris, M., Castellano, A., Furlanello, C., Locatelli, F., and Fruci, D. (2015). Tumor-infiltrating T lymphocytes improve clinical outcome of therapy-resistant neuroblastoma. OncoImmunology *4*, e1019981. https://doi.org/10.1080/2162402x.2015.1019981.
6. Wienke, J., Dierselhuis, M.P., Tytgat, G.A., Kunkele, A., Nierkens, S., and Molenaar, J.J. (2021). The immune landscape of neuroblastoma: challenges and opportunities for novel therapeutic strategies in pediatric oncology. Eur. J. Cancer *144*, 123–150. https://doi.org/10.1016/j.ejca.2020.11.014.
7. Ollé Hurtado, M., Wolbert, J., Fisher, J., Flutter, B., Stafford, S., Barton, J., Jain, N., Barone, G., Majani, Y., and Anderson, J. (2019). Tumor infiltrating lymphocytes expanded from pediatric neuroblastoma display heterogeneity of phenotype and function. PLoS One *14*, e0216373. https://doi.org/10.1371/journal.pone.0216373.
8. Facchetti, P., Prigione, I., Ghiotto, F., Tasso, P., Garaventa, A., and Pistoia, V. (1996). Functional and molecular characterization of tumour-infiltrating lymphocytes and clones thereof from a major-histocompatibility-complex-negative human tumour: Neuroblastoma. Cancer Immunol. Immunother. *42*, 170–178. https://doi.org/10.1007/s002620050267.
9. Valteau, D., Scott, V., Carcelain, G., Hartmann, O., Escudier, B., Hercend, T., and Triebel, F. (1996). T-cell receptor repertoire in neuroblastoma patients. Cancer Res. *56*, 362–369.
10. Wei, J.S., Kuznetsov, I.B., Zhang, S., Song, Y.K., Asgharzadeh, S., Sindiri, S., Wen, X., Patidar, R., Najaraj, S., Walton, A., et al. (2018). Clinically relevant cytotoxic immune cell signatures and clonal expansion of T-cell receptors in high-risk MYCN-not-amplified human neuroblastoma. Clin. Cancer Res. *24*, 5673–5684. https://doi.org/10.1158/1078-0432.ccr-18-0599.
11. Carlson, L.-M., De Geer, A., Sveinbjornsson, B., Orrego, A., Martinsson, T., Kogner, P., and Levitskaya, J. (2013). The microenvironment of human neuroblastoma supports the activation of tumor-associated T lymphocytes. OncoImmunology *2*, e23618. https://doi.org/10.4161/onci.23618.
12. Szabo, P.A., Miron, M., and Farber, D.L. (2019). Location, location, location: tissue resident memory T cells in mice and humans. Sci. Immunol. *4*, 1–12. https://doi.org/10.1126/sciimmunol.aas9673.
13. Hishiki, T., Mise, N., Harada, K., Ihara, F., Takami, M., Saito, T., Terui, K., Nakata, M., Komatsu, S., Yoshida, H., and Motohashi, S. (2018). Invariant natural killer T infiltration in neuroblastoma with favorable outcome. Pediatr. Surg. Int. *34*, 195–201. https://doi.org/10.1007/s00383-017-4189-x.
14. Melaiu, O., Chierici, M., Lucarini, V., Jurman, G., Conti, L.A., De Vito, R., Boldrini, R., Cifaldi, L., Castellano, A., Furlanello, C., et al. (2020). Cellular and gene signatures of tumor-infiltrating dendritic cells and natural-killer cells predict prognosis of neuroblastoma. Nat. Commun. *11*, 5992. https://doi.org/10.1038/s41467-020-19781-y.
15. Zhang, P., Wu, X., Basu, M., Dong, C., Zheng, P., Liu, Y., and Sandler, A.D. (2017). MYCN amplification is associated with repressed cellular immunity in neuroblastoma: an in silico immunological analysis of TARGET database. Front. Immunol. *8*, 1–9. https://doi.org/10.3389/fimmu.2017.01473.
16. Hashimoto, O., Yoshida, M., Koma, Y., Yanai, T., Hasegawa, D., Kosaka, Y., Nishimura, N., and Yokozaki, H. (2016). Collaboration of cancer-associated fibroblasts and tumour-associated macrophages for neuroblastoma development. J. Pathol. *240*, 211–223. https://doi.org/10.1002/path.4769.
17. Asgharzadeh, S., Salo, J.A., Ji, L., Oberthuer, A., Fischer, M., Berthold, F., Hadjidaniel, M., Liu, C.W.Y., Metelitsa, L.S., Pique-Regi, R., et al. (2012). Clinical significance of tumor-associated inflammatory cells in metastatic neuroblastoma. J. Clin. Oncol. *30*, 3525–3532. https://doi.org/10.1200/jco.2011.40.9169.
18. Cassetta, L., Fragkogianni, S., Sims, A.H., Swierczak, A., Forrester, L.M., Zhang, H., Soong, D.Y., Cotechini, T., Anur, P., Lin, E.Y., et al. (2019). Human tumor-associated macrophage and monocyte transcriptional landscapes reveal cancer-specific reprogramming, biomarkers, and therapeutic targets. Cancer Cell *35*, 588–602.e10. https://doi.org/10.1016/j.ccell.2019.02.009.
19. Kiss, M., Van Gassen, S., Movahedi, K., Saeys, Y., and Laoui, D. (2018). Myeloid cell heterogeneity in cancer: not a single cell alike. Cell. Immunol. *330*, 188–201. https://doi.org/10.1016/j.cellimm.2018.02.008.
20. Mao, Y., Eissler, N., Blanc, K.L., Johnsen, J.I., Kogner, P., and Kiessling, R. (2016). Targeting suppressive myeloid cells potentiates checkpoint inhibitors to control spontaneous neuroblastoma. Clin. Cancer Res. *22*, 3849–3859. https://doi.org/10.1158/1078-0432.ccr-15-1912.
21. Frosch, J., Leontari, I., and Anderson, J. (2021). Combined effects of myeloid cells in the neuroblastoma tumor microenvironment. Cancers *13*, 1743. https://doi.org/10.3390/cancers13071743.

22. Carlson, L.M., Rasmuson, A., Idborg, H., Segerstrom, L., Jakobsson, P.J., Sveinbjornsson, B., and Kogner, P. (2013). Low-dose aspirin delays an inflammatory tumor progression in vivo in a transgenic mouse model of neuroblastoma. Carcinogenesis *34*, 1081–1088. https://doi.org/10.1093/carcin/bgt009.

23. Voeller, J., and Sondel, P.M. (2019). Advances in anti-GD2 immunotherapy for treatment of high-risk neuroblastoma. J. Pediatr. Hematol. Oncol. *41*, 163–169. https://doi.org/10.1097/mph.0000000000001369.

24. Yu, A.L., Gilman, A.L., Ozkaynak, M.F., Naranjo, A., Diccianni, M.B., Gan, J., Hank, J.A., Batova, A., London, W.B., Tenney, S.C., et al. (2021). Long-term follow-up of a phase III study of ch14.18 (dinutuximab) + cytokine immunotherapy in children with high-risk neuroblastoma: COG study ANBL0032. Clin. Cancer Res. *27*, 2179–2189. https://doi.org/10.1158/1078-0432.ccr-20-3909.

25. Kameneva, P., Artemov, A.V., Kastriti, M.E., Faure, L., Olsen, T.K., Otte, J., Erickson, A., Semsch, B., Andersson, E.R., Ratz, M., et al. (2021). Single-cell transcriptomics of human embryos identifies multiple sympathoblast lineages with potential implications for neuroblastoma origin. Nat. Genet. *53*, 694–706. https://doi.org/10.1038/s41588-021-00818-x.

26. Kildisiute, G., Kholosy, W.M., Young, M.D., Roberts, K., Elmentaite, R., van Hooff, S.R., Pacyna, C.N., Khabirova, E., Piapi, A., Thevanesan, C., et al. (2021). Tumor to normal single-cell mRNA comparisons reveal a pan-neuroblastoma cancer cell. Sci. Adv. *7*, 1–14. https://doi.org/10.1126/sciadv.abd3311.

27. Dong, R., Yang, R., Zhan, Y., Lai, H.D., Ye, C.J., Yao, X.Y., Luo, W.Q., Cheng, X.M., Miao, J.J., Wang, J.F., et al. (2020). Single-cell characterization of malignant phenotypes and developmental trajectories of adrenal neuroblastoma. Cancer Cell *38*, 716–733.e6. https://doi.org/10.1016/j.ccell.2020.08.014.

28. Olsen, T.K., Otte, J., Mei, S., Kameneva, P., Björklund, A., Kryukov, E., Hou, Z., Johansson, A., Sundström, E., Martinsson, T., et al. (2020). Malignant Schwann cell precursors mediate intratumoral plasticity in human neuroblastoma. Preprint at bioRxiv. https://doi.org/10.1101/2020.05.04.077057.

29. Hanemaaijer, E.S., Margaritis, T., Sanders, K., Bos, F.L., Candelli, T., Al-Saati, H., van Noesel, M.M., Meyer-Wentrup, F.A.G., van de Wetering, M., Holstege, F.C.P., and Clevers, H. (2021). Single-cell atlas of developing murine adrenal gland reveals relation of Schwann cell precursor signature to neuroblastoma phenotype. Proc. Natl. Acad. Sci. U S A *118*. https://doi.org/10.1073/pnas.2022350118.

30. Bedoya-Reina, O.C., Li, W., Arceo, M., Plescher, M., Bullova, P., Pui, H., Kaucka, M., Kharchenko, P., Martinsson, T., Holmberg, J., et al. (2021). Single-nuclei transcriptomes from human adrenal gland reveal distinct cellular identities of low and high-risk neuroblastoma tumors. Nat. Commun. 12, 1–15.

31. Jansky, S., Sharma, A.K., Korber, V., Quintero, A., Toprak, U.H., Wecht, E.M., Gartlgruber, M., Greco, A., Chomsky, E., Grunewald, T.G.P., et al. (2021). Single-cell transcriptomic analyses provide insights into the developmental origins of neuroblastoma. Nat. Genet. *53*, 683–693. https://doi.org/10.1038/s41588-021-00806-1.

32. Kfoury, Y., Baryawno, N., Severe, N., Mei, S., Gustafsson, K., Hirz, T., Brouse, T., Scadden, E.W., Igolkina, A.A., Kokkaliaris, K., et al. (2021). Human prostate cancer bone metastases have an actionable immunosuppressive microenvironment. Cancer Cell, 1464–1478.e8.

33. Barkas, N., Petukhov, V., Nikolaeva, D., Lozinsky, Y., Demharter, S., Khodosevich, K., and Kharchenko, P.V. (2019). Joint analysis of heterogeneous single-cell RNA-seq dataset collections. Nat. Methods *16*, 695–698. https://doi.org/10.1038/s41592-019-0466-z.

34. Elizondo, D.M., Brandy, N.Z.D., da Silva, R.L.L., Haddock, N.L., Kacsinta, A.D., de Moura, T.R., and Lipscomb, M.W. (2019). Allograft inflammatory factor-1 governs hematopoietic stem cell differentiation into CDC1 and monocyte-derived dendritic cells through IRF8 and RelB in vitro. Front. Immunol. *10*, 1–13. https://doi.org/10.3389/fimmu.2019.00173.

35. Zilionis, R., Engblom, C., Pfirschke, C., Savova, V., Zemmour, D., Saatcioglu, H.D., Krishnan, I., Maroni, G., Meyerovitz, C.V., Kerwin, C.M., et al. (2019). Single-cell transcriptomics of human and mouse lung cancers reveals conserved myeloid populations across individuals and species. Immunity *50*, 1317–1334.e10. https://doi.org/10.1016/j.immuni.2019.03.009.

36. Yang, J., Ren, J., Yang, Y., Sun, J., Zhou, X., Zheng, S., Xuan, D., Xue, Y., Fan, H., Zhang, J., et al. (2018). BANK1 alters B cell responses and influences the interactions between B cells and induced T regulatory cells in mice with collagen-induced arthritis. Arthritis Res. Ther. *20*, 9–13. https://doi.org/10.1186/s13075-017-1503-x.

37. Leandro, M.J. (2013). B-cell subpopulations in humans and their differential susceptibility to depletion with anti-CD20 monoclonal antibodies. Arthritis Res. Ther. *15*, S3–S8. https://doi.org/10.1186/ar3908.

38. Szabo, P.A., Levitin, H.M., Miron, M., Snyder, M.E., Senda, T., Yuan, J., Cheng, Y.L., Bush, E.C., Dogra, P., Thapa, P., et al. (2019). Single-cell transcriptomics of human T cells reveals tissue and activation signatures in health and disease. Nat. Commun. *10*, 4706. https://doi.org/10.1038/s41467-019-12464-3.

39. Yang, C., Siebert, J.R., Burns, R., Gerbec, Z.J., Bonacci, B., Rymaszewski, A., Rau, M., Riese, M.J., Rao, S., Carlson, K.S., et al. (2019). Heterogeneity of human bone marrow and blood natural killer cells defined by single-cell transcriptome. Nat. Commun. *10*, 3931. https://doi.org/10.1038/s41467-019-11947-7.

40. Chen, J., Cheung, F., Shi, R., Zhou, H., and Lu, W. (2018). PBMC fixation and processing for Chromium single-cell RNA sequencing. J. Transl. Med. *16*, 198. https://doi.org/10.1186/s12967-018-1578-4.

41. Olingy, C.E., Dinh, H.Q., and Hedrick, C.C. (2019). Monocyte heterogeneity and functions in cancer. J. Leukoc. Biol. *106*, 309–322. https://doi.org/10.1002/jlb.4ri0818-311r.

42. DeNardo, D.G., and Ruffell, B. (2019). Macrophages as regulators of tumour immunity and immunotherapy. Nat. Rev. Immunol. *19*, 369–382. https://doi.org/10.1038/s41577-019-0127-6.

43. Villani, A.C., Satija, R., Reynolds, G., Sarkizova, S., Shekhar, K., Fletcher, J., Griesbeck, M., Butler, A., Zheng, S., Lazo, S., et al. (2017). Single-cell RNA-seq reveals new types of human blood dendritic cells, monocytes, and progenitors. Science *356*, eaah4573. https://doi.org/10.1126/science.aah4573.

44. Pombo Antunes, A.R., Scheyltjens, I., Lodi, F., Messiaen, J., Antoranz, A., Duerinck, J., Kancheva, D., Martens, L., De Vlaminck, K., Van Hove, H., et al. (2021). Single-cell profiling of myeloid cells in glioblastoma across species and disease stage reveals macrophage competition and specialization. Nat. Neurosci. *24*, 595–610. https://doi.org/10.1038/s41593-020-00789-y.

45. Xiong, D., Wang, Y., and You, M. (2020). A gene expression signature of TREM2hi macrophages and $\gamma\delta$ T cells predicts immunotherapy response. Nat. Commun. *11*, 5084. https://doi.org/10.1038/s41467-020-18546-x.

46. Cassetta, L., and Pollard, J.W. (2018). Targeting macrophages: therapeutic approaches in cancer. Nat. Rev. Drug Discov. *17*, 887–904. https://doi.org/10.1038/nrd.2018.169.

47. Palomino, D.C.T., and Marti, L.C. (2015). Chemokines and immunity. Einstein *13*, 469–473. https://doi.org/10.1590/s1679-45082015rb3438.

48. Krishna, C., DiNatale, R.G., Kuo, F., Srivastava, R.M., Vuong, L., Chowell, D., Gupta, S., Vanderbilt, C., Purohit, T.A., Liu, M., et al. (2021). Single-cell sequencing links multiregional immune landscapes and tissue-resident T cells in ccRCC to tumor topology and therapy efficacy. Cancer Cell *39*, 662–677.e6. https://doi.org/10.1016/j.ccell.2021.03.007.

49. Krzyzak, L., Seitz, C., Urbat, A., Hutzler, S., Ostalecki, C., Glasner, J., Hiergeist, A., Gessner, A., Winkler, T.H., Steinkasserer, A., and Nitschke, L. (2016). CD83 modulates B cell activation and germinal center responses. J. Immunol. *196*, 3581–3594. https://doi.org/10.4049/jimmunol.1502163.

50. King, H.W., Orban, N., Riches, J.C., Clear, A.J., Warnes, G., Teichmann, S.A., and James, L.K. (2021). Single-cell analysis of human B cell

maturation predicts how antibody class switching shapes selection dynamics. Sci. Immunol. *6*, eabe6291. https://doi.org/10.1126/sciimmunol.abe6291.

51. Helmink, B.A., Reddy, S.M., Gao, J., Zhang, S., Basar, R., Thakur, R., Yizhak, K., Sade-Feldman, M., Blando, J., Han, G., et al. (2020). B cells and tertiary lymphoid structures promote immunotherapy response. Nature *577*, 549–555. https://doi.org/10.1038/s41586-019-1922-8.

52. Laidlaw, B.J., and Cyster, J.G. (2021). Transcriptional regulation of memory B cell differentiation. Nat. Rev. Immunol. *21*, 209–220. https://doi.org/10.1038/s41577-020-00446-2.

53. Chi, X., Li, Y., and Qiu, X. (2020). V(D)V(D)J recombination, somatic hypermutation and class switch recombination of immunoglobulins: mechanism and regulation. Immunology *160*, 233–247. https://doi.org/10.1111/imm.13176.

54. Holmes, A.B., Corinaldesi, C., Shen, Q., Kumar, R., Compagno, N., Wang, Z., Nitzan, M., Grunstein, E., Pasqualucci, L., Dalla-Favera, R., and Basso, K. (2020). Single-cell analysis of germinal-center B cells informs on lymphoma cell of origin and outcome. J. Exp. Med. *217*, e20200483. https://doi.org/10.1084/jem.20200483.

55. Seifert, M., and Küppers, R. (2016). Human memory B cells. Leukemia *30*, 2283–2292. https://doi.org/10.1038/leu.2016.226.

56. Wu, J., and Lanier, L.L. (2003). Natural killer cells and cancer. Adv. Cancer Res. *90*, 127–156. https://doi.org/10.1016/s0065-230x(03)90004-2.

57. Melaiu, O., Lucarini, V., Cifaldi, L., and Fruci, D. (2020). Influence of the tumor microenvironment on NK cell function in solid tumors. Front. Immunol. *10*, 3038. https://doi.org/10.3389/fimmu.2019.03038.

58. Dogra, P., Rancan, C., Ma, W., Toth, M., Senda, T., Carpenter, D.J., Kubota, M., Matsumoto, R., Thapa, P., Szabo, P.A., et al. (2020). Tissue determinants of human NK cell development, function, and residence. Cell *180*, 749–763.e13. https://doi.org/10.1016/j.cell.2020.01.022.

59. Björklund, A.K., Forkel, M., Picelli, S., Konya, V., Theorell, J., Friberg, D., Sandberg, R., and Mjosberg, J. (2016). The heterogeneity of human CD127+ innate lymphoid cells revealed by single-cell RNA sequencing. Nat. Immunol. *17*, 451–460. https://doi.org/10.1038/ni.3368.

60. June, C.H., O'Connor, R.S., Kawalekar, O.U., Ghassemi, S., and Milone, M.C. (2018). CAR T cell immunotherapy for human cancer. Science *359*, 1361–1365. https://doi.org/10.1126/science.aar6711.

61. Zheng, C., Zheng, L., Yoo, J.K., Guo, H., Zhang, Y., Guo, X., Kang, B., Hu, R., Huang, J.Y., Zhang, Q., et al. (2017). Landscape of infiltrating T cells in liver cancer revealed by single-cell sequencing. Cell *169*, 1342–1356.e16. https://doi.org/10.1016/j.cell.2017.05.035.

62. Zou, W. (2006). Regulatory T cells, tumour immunity and immunotherapy. Nat. Rev. Immunol. *6*, 295–307. https://doi.org/10.1038/nri1806.

63. Zhang, Z., Liu, S., Zhang, B., Qiao, L., Zhang, Y., and Zhang, Y. (2020). T cell dysfunction and exhaustion in cancer. Front. Cell Dev. Biol. *8*. https://doi.org/10.3389/fcell.2020.00017.

64. Castro, G., Liu, X., Ngo, K., De Leon-Tabaldo, A., Zhao, S., Luna-Roman, R., Yu, J., Cao, T., Kuhn, R., Wilkinson, P., et al. (2017). RORγt and RORα signature genes in human Th17 cells. PLoS One *12*, e0181868. https://doi.org/10.1371/journal.pone.0181868.

65. Zhang, H., Chai, W., Yang, W., Han, W., Mou, W., Xi, Y., Chen, X., Wang, H., Wang, W., Qin, H., et al. (2020). The increased IL-17-producing $\gamma\delta$T cells promote tumor cell proliferation and migration in neuroblastoma. Clin. Immunol. *211*, 108343. https://doi.org/10.1016/j.clim.2020.108343.

66. Li, Z., Li, D., Tsun, A., and Li, B. (2015). FOXP3+ regulatory T cells and their functional regulation. Cell. Mol. Immunol. *12*, 558–565. https://doi.org/10.1038/cmi.2015.10.

67. Cibrián, D., and Sánchez-Madrid, F. (2017). CD69: from activation marker to metabolic gatekeeper. Eur. J. Immunol. *47*, 946–953. https://doi.org/10.1002/eji.201646837.

68. Bhat, P., Leggatt, G., Waterhouse, N., and Frazer, I.H. (2017). Interferon-γ derived from cytotoxic lymphocytes directly enhances their motility and cytotoxicity. Cell Death Dis. *8*, e2836. https://doi.org/10.1038/cddis.2017.67.

69. Zhong, X., Zhang, Y., Wang, L., Zhang, H., Liu, H., and Liu, Y. (2019). Cellular components in tumor microenvironment of neuroblastoma and the prognostic value. PeerJ *7*, e8017. https://doi.org/10.7717/peerj.8017.

70. Melaiu, O., Mina, M., Chierici, M., Boldrini, R., Jurman, G., Romania, P., D'Alicandro, V., Benedetti, M.C., Castellano, A., Liu, T., et al. (2017). PD-L1 is a therapeutic target of the bromodomain inhibitor JQ1 and, combined with HLA class I, a promising prognostic biomarker in neuroblastoma. Clin. Cancer Res. *23*, 4462–4472. https://doi.org/10.1158/1078-0432.ccr-16-2601.

71. Efremova, M., Vento-Tormo, M., Teichmann, S.A., and Vento-Tormo, R.C.P.D.B. (2020). CellPhoneDB: inferring cell–cell communication from combined expression of multi-subunit ligand–receptor complexes. Nat. Protoc. *15*, 1484–1506. https://doi.org/10.1038/s41596-020-0292-x.

72. Groningen, T. V., Akogul, N., Westerhout, E. M., Chan, A. & Hasselt, N. E. A NOTCH feed-forward loop drives reprogramming from adrenergic- to mesenchymal state in neuroblastoma.Nat. Commun. 10,.1530

73. Påhlman, S., Stockhausen, M.T., Fredlund, E., and Axelson, H. (2004). Notch signaling in neuroblastoma. Semin. Cancer Biol. *14*, 365–373. https://doi.org/10.1016/j.semcancer.2004.04.016.

74. Funke, L., Bracht, T., Oeck, S., Schork, K., Stepath, M., Dreesmann, S., Eisenacher, M., Sitek, B., and Schramm, A. (2021). NTRK1/TrkA signaling in neuroblastoma cells induces nuclear reorganization and intra-nuclear aggregation of lamin A/C. Cancers, 5293.

75. Chen, D.S., and Mellman, I. (2013). Oncology meets immunology: the cancer-immunity cycle. Immunity *39*, 1–10. https://doi.org/10.1016/j.immuni.2013.07.012.

76. Szanto, C.L., Cornel, A.M., Tamminga, S.M., Delemarre, E.M., de Koning, C.C.H., van den Beemt, D.A.M.H., Dunnebach, E., Tas, M.L., Dierselhuis, M.P., Tytgat, L.G.A.M., et al. (2021). Immune monitoring during therapy reveals activity and regulatory immune responses in high-risk neuroblastoma. Cancers *13*, 2096. https://doi.org/10.3390/cancers13092096.

77. Zitvogel, L., Apetoh, L., Ghiringhelli, F., and Kroemer, G. (2008). Immunological aspects of cancer chemotherapy. Nat. Rev. Immunol. *8*, 59–73. https://doi.org/10.1038/nri2216.

78. Liu, K.X., and Joshi, S. (2020). "Re-educating" tumor associated macrophages as a novel immunotherapy strategy for neuroblastoma. Front. Immunol. *11*, 1–17. https://doi.org/10.3389/fimmu.2020.01947.

79. Multhoff, G., Molls, M., and Radons, J. (2012). Chronic inflammation in cancer development. Front. Immunol. *2*, 1–17. https://doi.org/10.3389/fimmu.2011.00098.

80. Petitprez, F., de Reynies, A., Keung, E.Z., Chen, T.W.W., Sun, C.M., Calderaro, J., Jeng, Y.M., Hsiao, L.P., Lacroix, L., Bougouin, A., et al. (2020). B cells are associated with survival and immunotherapy response in sarcoma. Nature *577*, 556–560. https://doi.org/10.1038/s41586-019-1906-8.

81. Cabrita, R., Lauss, M., Sanna, A., Donia, M., Skaarup Larsen, M., Mitra, S., Johansson, I., Phung, B., Harbst, K., Vallon-Christersson, J., et al. (2020). Author Correction: tertiary lymphoid structures improve immunotherapy and survival in melanoma. Nature *580*, 561–565. https://doi.org/10.1038/s41586-020-2155-6.

82. Akinleye, A., and Rasool, Z. (2019). Immune checkpoint inhibitors of PD-L1 as cancer therapeutics. J. Hematol. Oncol. *12*, 92. https://doi.org/10.1186/s13045-019-0779-5.

83. Nishikawa, H., and Koyama, S. (2021). Mechanisms of regulatory T cell infiltration in tumors: implications for innovative immune precision therapies. J. Immunother. Cancer *9*, e002591. https://doi.org/10.1136/jitc-2021-002591.

84. Ross, R.A., Walton, J.D., Han, D., Guo, H.F., and Cheung, N.K.V. (2015). A distinct gene expression signature characterizes human neuroblastoma cancer stem cells. Stem Cell Res. *15*, 419–426. https://doi.org/10.1016/j.scr.2015.08.008.

85. Mathewson, N.D., Ashenberg, O., Tirosh, I., Gritsch, S., Perez, E.M., Marx, S., Jerby-Arnon, L., Chanoch-Myers, R., Hara, T., Richman, A.R., et al. (2021). Inhibitory CD161 receptor identified in glioma-infiltrating T cells by single-cell analysis. Cell *184*, 1281–1298.e26. https://doi.org/10.1016/j.cell.2021.01.022.

86. Nakamura, K., and Smyth, M.J. (2020). TREM2 marks tumor-associated macrophages. Signal Transduct. Target. Ther. *5*, 233. https://doi.org/10.1038/s41392-020-00356-8.

87. Molgora, M., Esaulova, E., Vermi, W., Hou, J., Chen, Y., Luo, J., Brioschi, S., Bugatti, M., Omodei, A.S., Ricci, B., et al. (2020). TREM2 modulation remodels the tumor myeloid landscape enhancing anti-PD-1 immunotherapy. Cell *182*, 886–900.e17. https://doi.org/10.1016/j.cell.2020.07.013.

88. Ndhlovu, L.C., Lopez-Verges, S., Barbour, J.D., Jones, R.B., Jha, A.R., Long, B.R., Schoeffler, E.C., Fujita, T., Nixon, D.F., and Lanier, L.L. (2012). Tim-3 marks human natural killer cell maturation and suppresses cell-mediated cytotoxicity. Blood *119*, 3734–3743. https://doi.org/10.1182/blood-2011-11-392951.

89. Khan, M., Arooj, S., and Wang, H. (2020). NK cell-based immune checkpoint inhibition. Front. Immunol. *11*, 167. https://doi.org/10.3389/fimmu.2020.00167.

90. Barkal, A.A., Brewer, R.E., Markovic, M., Kowarsky, M., Barkal, S.A., Zaro, B.W., Krishnan, V., Hatakeyama, J., Dorigo, O., Barkal, L.J., and Weissman, I.L. (2019). CD24 signalling through macrophage Siglec-10 is a target for cancer immunotherapy. Nature *572*, 392–396. https://doi.org/10.1038/s41586-019-1456-0.

91. Yarmarkovich, M., Marshall, Q.F., Warrington, J.M., Premaratne, R., Farrel, A., Groff, D., Li, W., Marco, M., Runbeck, E., Truong, H., et al. (2021). Cross-HLA targeting of intracellular oncoproteins with peptide-centric CARs. Nature, 477–484. https://doi.org/10.1038/s41586-021-04061-6.

STAR★METHODS

KEY RESOURCES TABLE

REAGENT or RESOURCE	SOURCE	IDENTIFIER
Antibodies		
Anti-CD235-PE	Biolegend	306603/306604; RRID: AB_314621
CD3 (UCHT1)	Akoya	4350008; RRID: AB_2895047
CD4 (SK3)	Akoya	4350010; RRID: AB_2895048
CD8 (SK1)	Akoya	4150004; RRID: AB_2895049
CD11c (S-HCL-3)	Akoya	4350012; RRID: AB_2895050
CD19 (HIB19)	Akoya	4350003; no RRID available
CD45RO (UCHL1)	Akoya	4150023; RRID: AB_2895053
HLA-DR (L243)	Akoya	4250006; RRID: AB_2895054
Ki67 (B56)	Akoya	4250019; RRID: AB_2895046
CODEX Nuclear Stain (SKU)	Akoya	7000003
Anti-CD68	Agilent	M0814 clone KP1; RRID: AB_2750584
Anti-CD3	Abcam	Ab16669 clone SP7; RRID: AB_443425
Anti-CD8	Agilent	M710301-2 clone C8/144B; RRID: AB_2075537
Anti-FOXP3	Abcam	Ab20034 clone 236A/E7; RRID: AB_445284
Anti-IL17	R&D Systems	AF-317-NA; RRID: AB_354463
Anti-NE	Abcam	Ab68672; RRID: AB_1658868
Anti-NKp46	R&D Systems	Clone 195314; RRID: AB_2149153
Anti-CD1a	Abcam	EP3622; RRID: AB_10864235
Biological samples		
Human neuroblastoma tumor samples for scRNAseq	Karolinska Institutet/Karolinska University Hospital	N/A
Human neuroblastoma tumor samples for IHC	Skåne University hospital biobank/Lund University hospital	N/A
Chemicals, peptides, and recombinant proteins		
Media 199	Thermofisher Scientific	Cat#12350039
DMEM	Thermofisher Scientific	Cat#15-013-CV
RPMI-1640	Thermofisher Scientific	Cat#11875101
Fetal Bovine Serum (FBS)	Life Technologies	Cat#A31605-01
Penicillin-Streptomycin	Life Technologies	Cat#15140-122
Collagenase type I	Worthington	Cat#LS004214
Collagenase type II	Worthington	Cat#LS004202
Collagenase type III	Worthington	Cat#LS004206
Collagenase type IV	Worthington	Cat#LS004210
Dispase 1	Thermofisher Scientific	Cat#17105041
RNasin Ribonuclease Inhibitor)	Promega	Cat#N2111
RNAase Out Recombinant Ribonuclease Inhibitor	Thermofisher Scientific	Cat#10777019
ACK-lysis buffer	Thermofisher Scientific	Cat#A1049201
Calcein AM	Thermofisher Scientific	Cat#C3099
7-AAD	Thermofisher Scientific	Cat#00-6993-50
DAPI	Thermofisher Scientific	Cat#62248
Ultrapure BSA	Thermofisher Scientific	Cat#AM2616
4% paraformaldehyde	Thermofisher Scientific	Cat#28908

(*Continued on next page*)

Continued

REAGENT or RESOURCE	SOURCE	IDENTIFIER
Phosphate Buffered Saline (PBS)	Thermofisher Scientific	Cat#14080048
PBS + 30%sucrose	Sigma	Cat#0389
OCT	VWR	Cat#00411243
Xylene	28973.294	VWR
1% H_2O_2	1.07209.0250	Merck
Target Retrieval Solution (DAKO)	DAKO	S1699
Protein Block Serum-Free Solution	DAKO	X0909
PBS + 5% normal goat serum	005-000-001<br>SH30256.01	Jackson ImmunoResearch<br>Nordic Biolabs
Labeled-chromogen substrate solution with Liquid DAB chromogen	DAKO	K3467
Mayer htx	HISTOLAB	01820
Faramount Aqueous Mounting Medium	DAKO	S3025
Critical commercial assays		
Chromium Single Cell 3′v2 and v3 Reagent Kit	10X Genomics	N/A
CODEX Staining Kit	Akoya Biosciences	Cat# 7000008
CODEX Assay Reagents	Akoya Biosciences	Cat # 7000002
10X CODEX Buffer	Akoya Biosciences	Cat # 7000001
Deposited data		
Data files for scRNA-seq	This paper	GEO: GSE147766
Software and algorithms		
Cellranger v2.0	10x Genomics	https://support.10xgenomics.com/single-cell-gene-expression/software/downloads/latest
Conos 1.2.1	Barkas et al., 2019	https://github.com/kharchenkolab/conos
InferCNV	Patel et al., 2014	https://github.com/broadinstitute/inferCNV
ClusterProfiler 4.0 R package	Wu et al., 2021	https://bioconductor.org/packages/release/bioc/html/clusterProfiler.html
Candisc 0.8-5 R package	Comprehensive R Archive Network (CRAN)	https://cran.r-project.org/web/packages/candisc/index.html
Survival 3.2-3 R package	Comprehensive R Archive Network (CRAN)	https://cran.r-project.org/web/packages/survival/index.html
R v3.6.0	https://www.r-project.org/	https://www.r-project.org/
Prism software	GraphPad	https://www.graphpad.com/scientific-%20software/prism/
CODEX Instrument Management Software	Akoya Biosciences	NA
Ks R package (for kernel density)	Comprehensive R Archive Network (CRAN)	https://cran.r-project.org/web/packages/ks/index.html
Other		
BD FACS Aria III	BD Biosciences	N/A
Inverted fluorescent microscope Leica Dmi8	N/A	N/A
Akoya CODEX instrument	Akoya Biosciences	N/A
Olympus BX43 microscope	Leica Biosystems	N/A

RESOURCE AVAILABILITY

Lead contact

Further information and requests for resources and reagents should be directed to and will be fulfilled by the lead contact, Ninib Baryawno (n.baryawno@ki.se).

Material availability

Further information and requests for resources and reagents should be directed to and will be fulfilled by the lead contact, Ninib Baryawno (n.baryawno@ki.se).

Data and code availability

- The expression datasets generated in this study are available through Gene Expression Omnibus with the accession number GEO: GSE147766.
- Interactive views of the single cell datasets, differential expression results, code notebooks, cell annotation and RData objects are available on the author's website at https://github.com/shenglinmei/NB.immune.atlas/.
- Any additional information required to reanalyze the data reported in this paper is available from the lead contact upon request.

EXPERIMENTAL MODEL AND SUBJECT DETAILS

Patient material and collection of tumor specimens

NB samples from Swedish patients were collected in conjunction with routine clinical sampling after parents and/or legal guardians had given verbal or written consent. All samples were collected according to permits approved by the Karolinska Institutet/Karolinska University Hospital ethics committees (reference numbers 03-736, 2009/1369-31/1) in accordance with the Helsinki declaration. Clinical management and therapy were performed according to national and international protocols and data were obtained from hospital records.

Tumor material used for immunohistochemistry

43 patients between the age 0 and 11 years were included in this study after written consent from their legal guardians. All patients have been treated at Lund University hospital from the year 2000 and forward. The tumor samples were accessed through the biobank at department of clinical genetics and pathology at Skåne University hospital and regional pathology archives and consists of multiple, formalin-fixed paraffin embedded (FFPE) tumor samples.

Ethical approval for material used in immunohistochemistry

All tissue samples and genetic data used during this project were collected from human tumors which were collected with informed consent from the legal guardians of the patients. Privacy of the donors have been protected and ethical approval from the ethical committee at Lund University has been obtained before the start of this project.

METHOD DETAILS

Dissociation of NB into single cells

Fresh tissue obtained from surgery was collected in Media 199 supplemented with 2% (v/v) FBS on ice. Single cell suspensions of the tumors were obtained by cutting the tumor into small pieces (1mm^3) followed by enzymatic dissociation for 45 minutes at 37°C with shaking at 120 rpm using Collagenase I, Collagenase II, Collagenase III, Collagenase IV (all at a concentration of 1 mg/mL) and Dispase (2 mg/mL) in the presence of DNAse I solution (1:100) and RNase Out (1:1000). Cells were then resuspended in fetal bovine serum (FBS) with 5% DMSO and cryopreserved in liquid nitrogen.

FACS enrichment of viable NB cells

Cryopreserved cells from NB samples were thawed and stained with anti-CD235-PE and Calcein AM for 30 minutes at 4°C. Cells were washed twice with Media199 containing 2% FBS followed by DAPI and/or 7AAD staining (1 μg/mL). Flow sorting for live and non-erythroid cells (DAPI/7AAD-negative, Calcein AM-positive, CD235-negative) was performed on a BD FACS Aria III equipped with a 100μm nozzle instrument.

Single cell library preparation and sequencing

After FACS sorting, NB single-cell libraries were prepared using the Chromium single-cell 3′ reagent kit v2 and v3 according to the manufacturer's recommendations. Libraries were sequenced on the Illumina NextSeq 500 platform.

Sectioning of NB tumors

Snap frozen NB samples were slowly brought from – 80°C to +4°C. Samples were fixed in 4% paraformaldehyde in PBS (pH 7.4) at 4°C for 4 hours with constant rotation, rinsed in PBS 3 times and cryoprotected by incubating at 4°C overnight in PBS containing 30% sucrose, again with constant rotation. Tissue samples were then embedded in OCT and frozen at −20°C. Serial sections (10, 14 or 40 μm) were produced from each sample and collected on SuperFrost Plus Adhesion microscope slides and stored at −20°C.

Highly multiplexed immunofluorescence

For highly multiplexed immunofluorescence of tissue sections with the CODEX (Co-detection by inDEXing) we used the service provided by the Spatial Proteomics Facility at Scilifelab. We used the automated image acquisition with an inverted fluorescent microscope, Leica Dmi8, and fluidics exchange was performed using an Akoya CODEX instrument and CIM (CODEX Instrument Management software). Staining was done for the following targets: CD3, CD4, CD8, CD11c, CD45RO, CD19, HLA-DR and Ki67. A nuclear stain was used as a reference marker between the different cycles (SKU 7000003 CODEX Nuclear Stain).

Immunohistochemistry

Immunohistochemistry was performed on 4 μm tissue sections from FFPE tumor samples. The slides were dried for 20 min, then deparaffinized in Xylene and rehydrated in ethanol to water. Endogenous peroxidase was blocked for 20 min with 1% H2O2. The slides were then washed, and epitope retrieval was performed using Target Retrieval Solution (DAKO) in 95 °C in a pressure cooker heater for 20 min. Slides were blocked in Protein Block Serum-Free Solution (X0909, DAKO) and later incubated with a primary antibody diluted in PBS containing 5% normal goat serum. Following incubation with primary antibody slides were washed in PBS and incubated with a secondary antibody. Labeled-chromogen substrate solution with Liquid DAB chromogen was applied. The slides were counterstained in Mayer htx before rehydrated and mounted with Faramount Aqueous Mounting Medium (S3025, DAKO).

The following primary antibodies were used: anti-CD68 (M0814, clone KP1, DAKO), anti-CD3 (ab16669, clone SP7, Abcam), anti-CD8 (M710301-2, clone C8/144B, Agilent), anti-FOXP3 (ab20034, clone 236A/E7, Abcam), anti-IL17 (AF-317-NA, R&D Systems), anti-NE (ab68672, Abcam), anti-NKp46 (clone 195314, R&D Systems) and anti-CD1a (EP3622, Ventana).

Infiltration density of immune cells in human NB

The infiltration density of immune cells in the tumors were evaluated by counting cells in 4 μm tissue sections from FFPE tumor samples. The FFPE samples had been stained using immunohistochemistry for the cell markers; CD68, CD3, CD8, FOXP3, IL17, NE, NKp46 and CD1a. 100 cells were counted in three different areas in each sample at 40x magnification (Olympus BX43 microscope, Olympus, Shinjuku, Japan) and the average percentage of infiltrating cells were calculated for each sample.

QUANTIFICATION AND STATISTICAL ANALYSIS

Pre-processing of scRNA-seq data

Demultiplexing of bcl files into fastq files was performed using *cellranger* 3.0.1 *mkfastq* software and alignments to the human genome reference sequences were performed using *cellranger count*. The reference included all protein coding genes as well as mitochondrial genes for downstream analysis. All cell barcodes with less than 500 UMIs were excluded. These were further filtered one sample at a time where barcodes with percent mitochondrial reads larger than median plus two standard deviations (percent.mito > median + 2sd). Likewise, barcodes with few detected genes were filtered out as $\log_{10}$(nGene) < median - 2sds.

ScRNA-seq analysis

During the preliminary round of analyses the datasets were aligned using *Conos* 1.2.1 with $k = 15,\ kself = 5$ PCA rotation space and angular distance measure. Visualization using UMAP embedding showed a number of continuous bridges connecting the major populations.

For subtype assessment within myeloid, T, NK or B cells, we extracted all myeloid (or T, NK, B cells), removed low quality samples with less than 50 cells per cells, and realigned separately using *Conos* with default paramenters. Leiden community clustering method (as implemented in *Conos*) was used to determine refined joint clusters, providing higher resolution than the initial analysis. In addition, we also provided walktrap.community and multilevel.community result in data website (https://github.com/shenglinmei/NB.imzmune.atlas), where users can download and view different clustering results. To avoid over clustering, we also evaluated cell type or cell states specific expressed genes. Final cell annotations are annotated based on literature reported markers and cluster specific markers (Table S5). getDifferentialGenes function from Conos was used to identify differentially expressed (marker) genes for clusters or subtypes. Annotation of the cluster communities was done using marker gene expression. Two patient samples were excluded for immune cell analysis due to low number of immune cells. These samples were included in the receptor ligand analysis where tumor and stroma are taken into account (see section Identification of significant ligand-receptor pairs).

Analysis of cell composition shift

We present two methods to analysis relative cell compositions shift within the major cell populations. The first one is cluster-based cell proportion analysis, cell frequency is normalized by total number of cells per sample (like total myeloid cells), statistical significance of proportion differences was then evaluated using Wilcoxon rank sum test. The second one is cluster-free cell composition analysis, where we estimate cell density per sample and evaluate the differential cell density between sample groups. We first compute kernel density in joint embedding space for each sample using *ks* R package (bin = 400), then quantile normalization was used to normalize the density matrix across samples and the average density of each sample group is shown in Figure 2H.

Calculation of gene set signature scores

To assess cell states in different cell subsets and conditions, we used a gene set signature score to measure the relative difference of cell states. The signature scores were calculated as average expression values of the genes in a given set. Specifically, we first calculated signature score for each cell as an average normalized (for cell size) gene expression magnitudes, then the signature score for each sample was computed as the mean across all cells.

Survival analysis

We collected 498 bulk RNA sequencing data of primary NB patient samples from GSE49711 (https://www.ncbi.nlm.nih.gov/geo/query/acc.cgi?acc=GSE49711). To test if a given signature predicts survival of NB patient, we first computed the average gene expression level of the signature in each tumor based on the bulk RNA sequencing data. The samples were grouped into high (top 25%) and low (bottom 25%) groups based on the average signature gene expression. Next, we used a one-sided log-rank test to compute the significance of the association between the signature expression value and prognosis. To evaluate the satiability of signature genes list, we resample the signature genes and repeat the analysis with 500 bootstrap resampling rounds. Statistical significance was then assessed by p-values at the 99% reproducibility power (i.e. reporting 0.99th quantile of the sampled p-values). Similarly, the same survival analysis was separately applied to low-risk and high-risk patients, MYCN amplified and MYCN non-amplified patients. The detailed genes list used for prognostic analysis can be found in Table S5. For survival heatmap plots, P-value from the survival plot was taken and presented as heatmap using -log10 P-value.

Identification of significant ligand-receptor pairs

The ligand-receptor (LR) pairs were downloaded from published databases *CellPhoneDB* (v1.1.0). To identify significant ligand-receptor pairs in 10X data (n = 19 patient samples), we used a similar approach as previously described (Vento-Tormo et al., 2018). We first calculated gene expression ratio in each cell type and only considered genes with more than 10% of cells demonstrating expression within each cell type. We then calculated average expression of ligand and receptor pairs across cell type pairs in normalized scRNA-seq data. The product of average ligand expression in cell type A and the average receptor expression in cell type B was used to measure LR pair expression. Statistical significance was assessed by randomly shuffling the cluster labels of all cell types and re-calculating ligand-receptor average pair expression across 1,000 permutations, which generated a null distribution for each LR pair in each pairwise comparison between two cell types. P-values were calculated with the normal distribution curve generated from the permuted LR pair interaction scores. Candidate LR pairs were determined by P value 0.05 or lower. To prioritize the significant ligand-receptor pairs, we tested if a LR pair is associated with patient overall survival. We first computed the average gene expression of the ligand and receptor in each tumor based on the bulk RNA-Seq data, next patients were stratified into two groups according to the average expression of the RL pair: high or low expression correspond to the top or bottom 25% of the population, respectively. Log-rank test was used to examine if there was a significant difference between these two patient groups in terms of their survival. LR pairs were filtered using survival significance p value of 0.05 or lower. In addition, we also evaluated ligand and receptor expression, requiring both ligand and receptor highly expressed in corresponding cell type. The *getDifferentialGenes()* function from Conos was used to derive DEG from each cell type and genes. We next screened each of the LR pair using p-value determined Z score >5 and AUC > 0.5. The detailed ligand-receptor pairs can be found at: https://github.com/shenglinmei/NB.immune.atlas.

Statistics

Wilcoxon rank sum test was used to assess significance in signature score analysis and cell proportion differences analysis. The p-values in the figures were reported using the following symbols: *p<0.05, **p<0.01, ***p<0.001, ****p<0.0001.

Data and code availability

Raw sequencing and processed data in this paper are available under the accession number: GSE147766. For the joint alignment analysis with public scRNA-seq data, we downloaded normal fetal adrenal tissue and NB scRNA-seq datasets from Dong et al., 2020, Kameneva et al., 2021 and Kildisiute et al., 2021. Custom code and the combined data that was used in this study can be found at https://github.com/shenglinmei/NB.immune.atlas. In addition, we provided an interactive view Conos object, allowing the user to download and view the data to the Conos joint alignment results.

Cell Reports Medicine, Volume *3*

Supplemental information

The immune cell atlas of human neuroblastoma

Bronte Manouk Verhoeven, Shenglin Mei, Thale Kristin Olsen, Karin Gustafsson, Anders Valind, Axel Lindström, David Gisselsson, Shahrzad Shirazi Fard, Catharina Hagerling, Peter V. Kharchenko, Per Kogner, John Inge Johnsen, and Ninib Baryawno

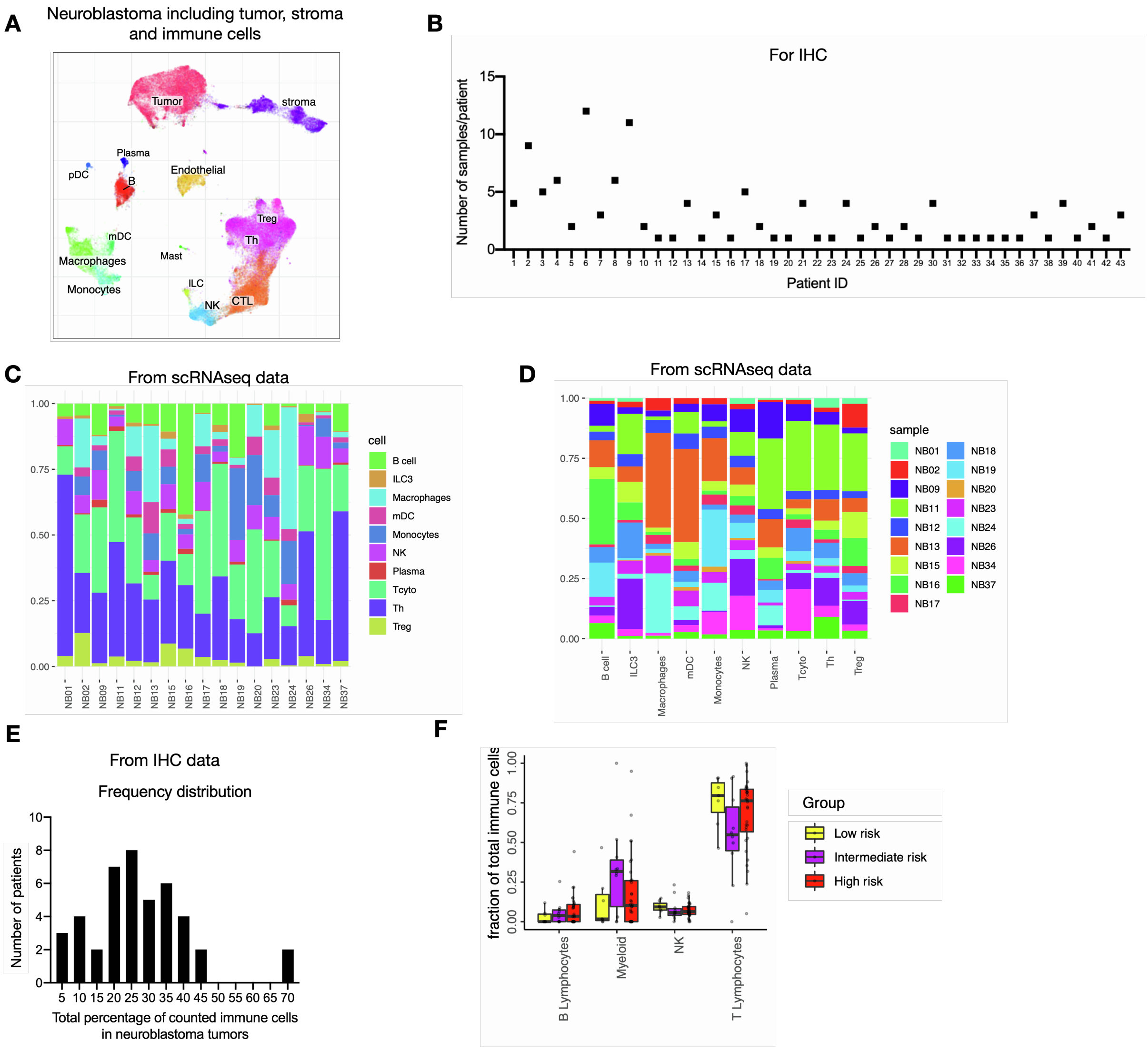

Figure S1. Global immune cell landscape of human NB. Related to Figure 1. (**A**) Global overview of neuroblastoma landscape including tumor and stromal cells from 19 patient samples. (**B**) The number of samples taken per patient used for the immunohistochemistry analysis. (**C**) The number of the different immune cell populations per patient sample (n=17) (from scRNAseq data). (**D**) Barplot showing cell composition of different immune cell types per patient sample (from scRNAseq data). (**E**) Frequency distribution of total percentage of counted immune cells in neuroblastoma tumors (from IHC data) shown as barplot (n=43). (**F**) Fraction of immune cell populations in low- (n=8), intermediate- (n=10) and high-risk (n=34) patient samples from the combined dataset including Kildisiute et al. Science Advances 2021, Dong et al. Cancer Cell 2020, Kameneva et al. Nature Genetics 2021 and the data presented here.

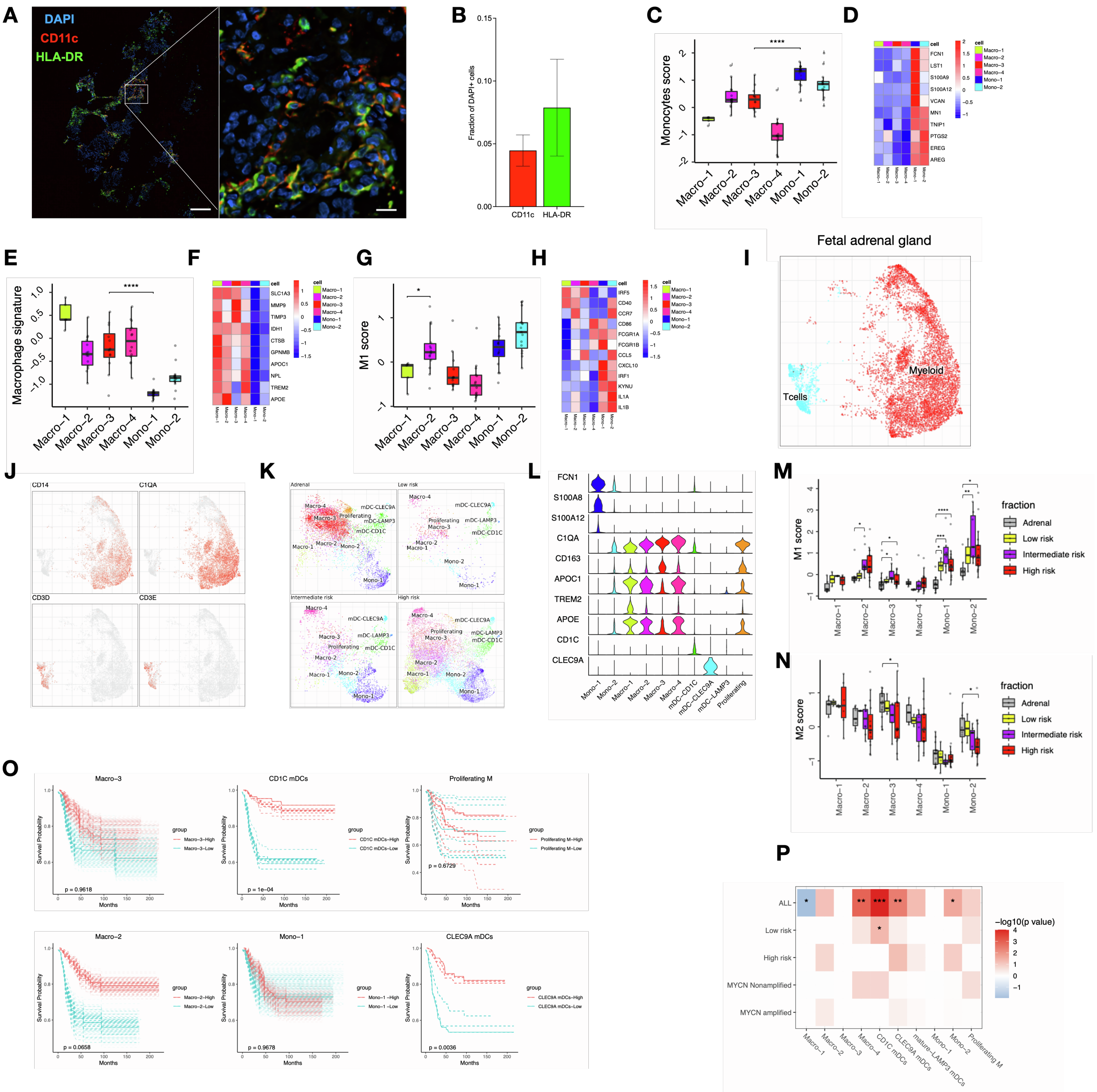

Figure S2. Myeloid cell infiltration with distinct cell-states detected in NB. Related to Figure 2. (**A**) Infiltration of CD11c+ myeloid cells and antigen presentation function (HLA-DR+) into neuroblastoma tumor using multiplex immunohistochemistry. Scale bar left = 800 µm, scale bar right = 50 µm. Picture taken from patient sample NB06. (**B**) Quantification for panel A presented as mean with standard error of the mean (n=3). (**C-D**) Average expression of monocyte score signature and accompanying heat map (n=16) (top color bar, colors matching panel C). Wilcoxon rank sum test was used for statistical analysis, *p<0.05, **p<0.01, ***p<0.001, ****p<0.0001. (**E-F**) Average expression of macrophage signature genes in different myeloid subpopulations and accompanying heat map (n=16) (top color bar, colors matching panel E). Wilcoxon rank sum test was used for statistical analysis, *p<0.05, **p<0.01, ***p<0.001, ****p<0.0001. (**G-H**) Average expression of M1 signature genes in different myeloid subpopulations and accompanying heat map (n=16) (top color bar, colors matching panel G). Wilcoxon rank sum test was used for statistical analysis, *p<0.05. (**I**) UMAP visualization of fetal adrenal immune cell data. (**J**) Accompanying key marker genes for I. (**K**) Myeloid cell specific embedding shows different numbers of different myeloid cell subpopulations comparing low- (n=5), intermediate- (n=8) and high-risk (n=21) neuroblastoma. (**L**) Violin plot displaying specific gene expression per population. Average expression of M1 (**M**) and M2 (**N**) signature genes in the combined dataset (see K for biological replicate numbers). Wilcoxon rank sum test was used for statistical analysis, *p<0.05, **p<0.01, ***p<0.001, ****p<0.0001. (**O**) Similar to Figure 1H, survival curves for Macro-2, Macro-3, Mono-1, CD1C mDCs, mature-LAMP3 mDCs, CLEC9A mDCs and Proliferating myeloid cells. (**P**) Summary of survival correlation split into risk group and MYCN status for the myeloid subtypes. Log-rank test was used for statistical analysis, *p<0.05, **p<0.01, ***p<0.001, ****p<0.0001.

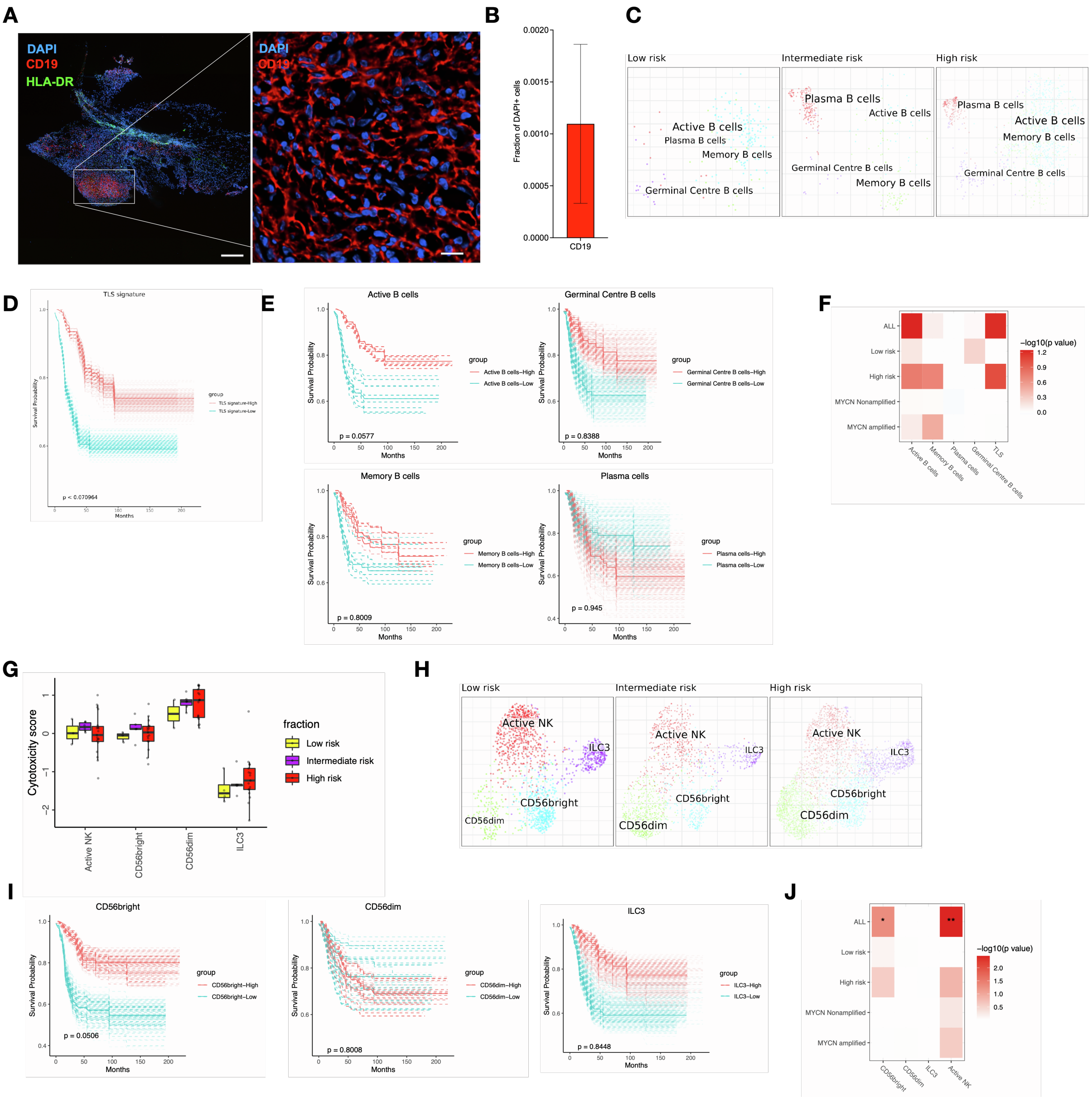

Figure S3. B and NK cell heterogeneity and infiltration in NB tumors. Related to Figure 3. (**A**) B cell (CD19+) and antigen presentation (HLA-DR+) staining using multiplex immunohistochemistry. Scale bar left = 1600 µm, scale bar right = 50 µm. Picture taken from patient sample NB11. (**B**) Quantification for panel A presented as mean with standard error of the mean (n=4). (**C**) B cell specific embedding shows different numbers of different B cell subpopulations comparing low- (n=3), intermediate- (n=6) and high-risk (n=18) neuroblastoma. (**D**) Similar to Figure 1H, survival curve for TLS signature. (**E**) Survival curves for the different B cell subpopulations. (**F**) Summary of survival correlation split into risk group and MYCN status for the B cell subtypes. Log-rank test was used for statistical analysis, *p<0.05, **p<0.01, ***p<0.001, ****p<0.0001.(**G**) Cytotoxicity signature score for the different NK cell populations comparing low- (n=4), intermediate- (n=6) and high-risk (n=21) disease. (**H**) The fraction of NK cells in low- (n=4), intermediate- (n=6) and high-risk (n=21) neuroblastoma on an NK cell specific embedding. (**I**) Survival curves for CD56bright, CD56dim and ILC3 cells. (**J**) Summary of survival correlation split into risk group and MYCN status for the NK cell subtypes. Log-rank test was used for statistical analysis, *p<0.05, **p<0.01, ***p<0.001, ****p<0.0001.

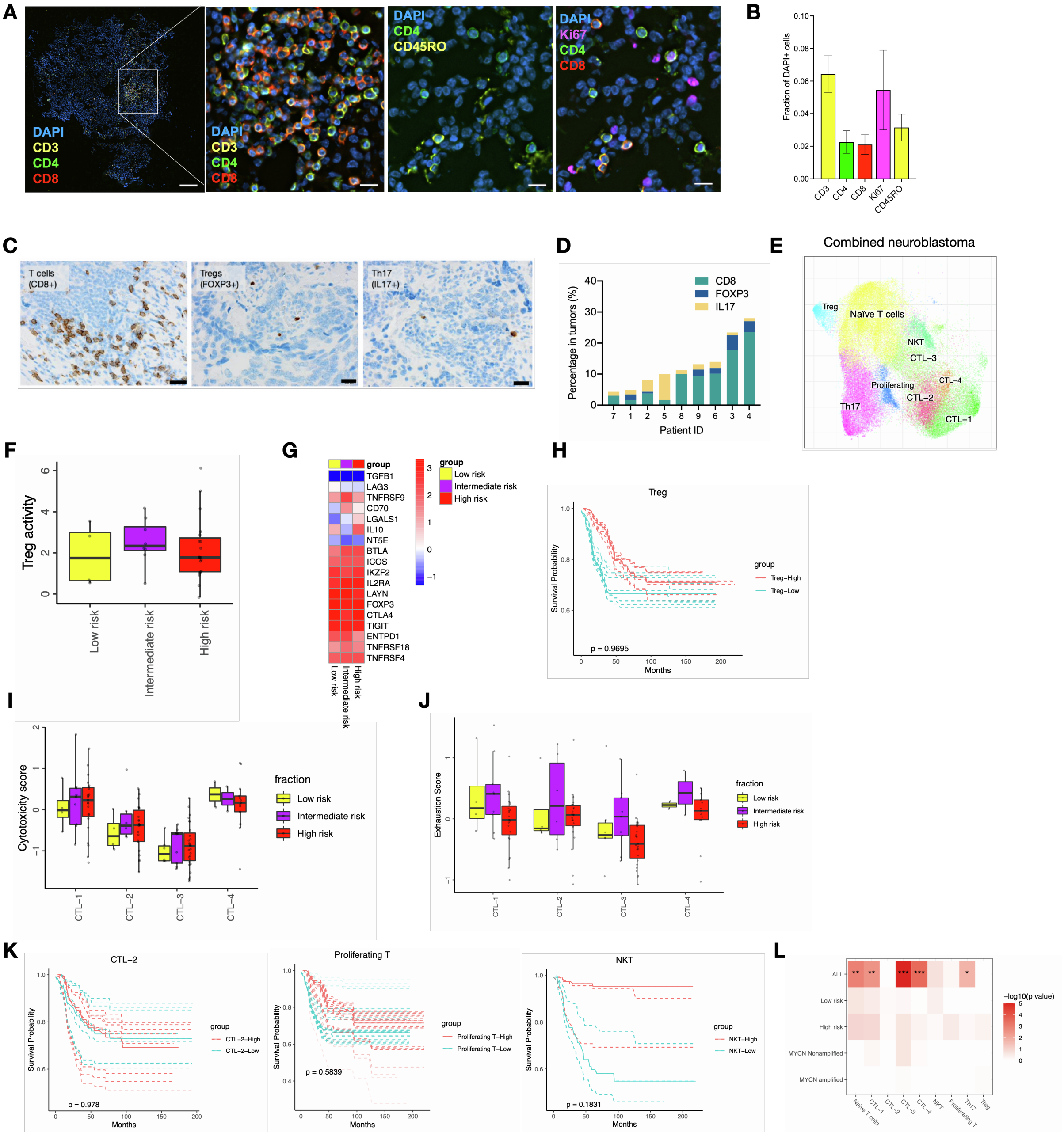

Figure S4. Distinct subtypes of T cell infiltrates correlate with improved NB survival. Related to Figure 4. (**A**) Multiplex IHC validation of different T cell populations in human neuroblastoma. Scale bar left = 800 µm, further right scale bars all are 50 µm. Staining included CD3+, CD4+, CD8+, memory cells (CD45RO+) and proliferating cells (Ki67+), and (**B**) quantification is presented as mean with standard error of the mean (n=3 to n=5). Pictures taken from patient sample NB01. (**C-D**) Additional single staining for CD8, FOXP3 and IL-17, and quantifications (n=9). (**E**) T cell specific embedding for the T cells present in the combined dataset (as used for the myeloid cell analysis). (**F**) Treg activity signature score comparing low- (n=4), intermediate- (n=9) and high-risk (n=23) disease with accompanying heat map (**G**). (**H**) Survival curve for Tregs. (**I-J**) Cytotoxicity and exhaustion signature score comparing low- (n=4), intermediate- (n=9) and high-risk (n=23) neuroblastoma for CTL-1 to -4. (**K**) Survival curves for CTL-2, proliferating T cells and NKT cells. (**L**) Summary of survival correlation split into risk group and MYCN status for the T cell subtypes. Log-rank test was used for statistical analysis, *p<0.05, **p<0.01, ***p<0.001, ****p<0.0001.

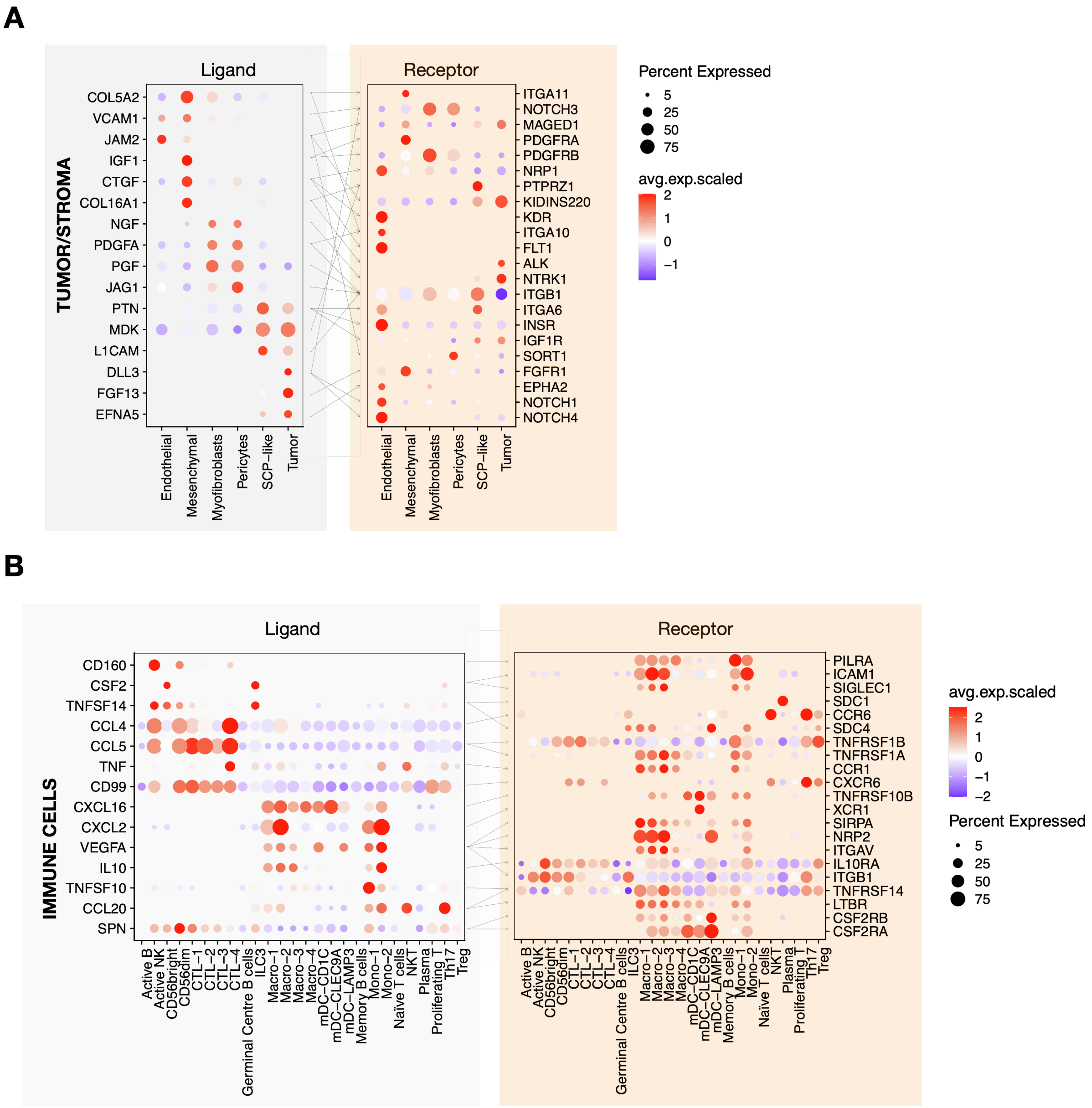

Figure S5. Tumor-immune cell ligand-receptor interaction analysis reveals several interactions for future studies. Related to Figure 5. (**A**) Similar to Figure 5C, showing expression of Ligand (tumor/stroma) - Receptor (tumor/stroma) interactions (n=19). (**B**) Ligand (immune) - Receptor (immune) interactions between immune cell subsets. The color represents scaled average expression of marker genes in each cell type, and the size indicates the proportion of cells expressing marker genes. Significance of ligand receptor pair is determined by permutation test, correlation to survival and specific cellular expression (see method).
